# Separation of Surface Grafted Microparticles via Light and Temperature

**DOI:** 10.1002/smsc.202400146

**Published:** 2024-08-13

**Authors:** Daniela Vasquez‐Muñoz, Fabian Rohne, Isabel Meier, Cevin Braksch, Nino Lomadze, Anahita Heraji Esfahani, Anne Nitschke, Andreas Taubert, Svetlana Santer, Matthias Hartlieb, Marek Bekir

**Affiliations:** ^1^ Institute of Physics and Astronomy University of Potsdam Karl‐Liebknecht‐Str. 24‐25 14476 Potsdam Germany; ^2^ Institute of Chemistry University of Potsdam Karl‐Liebknecht‐Str. 24‐25 14476 Potsdam Germany; ^3^ Fraunhofer Institute for Applied Polymer Research (IAP) Geiselbergstraße 69 14476 Potsdam Germany

**Keywords:** active colloids, azobenzene‐containing surfactants, microfluidics, PI‐RAFT polymerization, polymer brushes, volume phase transition temperature

## Abstract

Separation of equally sized particles distinguished solely by interfacial properties remains a highly challenging task. Herein, a particle fractioning method is proposed, which is suitable to differentiate between polymer‐grafted microparticles that are equal in size. The separation relies on the combination of a pressure driven microfluidic flow, together with simultaneous light illumination and temperature control. Heating the solution forces thermo‐responsive surface grafts to undergo a volume phase transition and therefore locally changing the interfacial properties of the microparticles. Light illumination induces the phoretic/osmotic activity of the microparticles and lifts them into a higher plane, where hovering particles experience a different shear stress proportional to the height. The light‐induced hovering height depends on the interfacial properties, and this complex interaction leads to different movements of the microparticles as a function of their surface grafting. The concepts are visualized in experimental studies, where the complex physical principle provides a simple method for fractioning a binary mixture with at least one thermo‐responsive polymer graft.

## Graphical abstract

A method for separating polymer‐grafted microparticles via distinguished differences in grafted polymer type and surface functionalization is demonstrated. The separation relies on differences in the velocity of the particles along a fluid flow induced by the combination of microfluidics, light, and temperature, where particles are dispersed in a photosensitive surfactant solution.

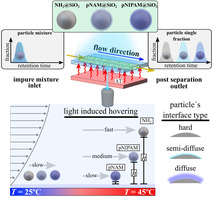

## 1 Introduction

Surface functionalization becomes a tool of paramount importance for the design of smart materials currently required for modern applications and technologies. In most cases this includes the chemical treatment of an interface via functional moieties, ligands, or polymers. In this way one can control affinity of an interface toward a solvent,^[^ ^1^, ^2^, ^3^, ^4^ ^]^ provide response to external stimuli via chemical gradients (pH, salt),^[^ ^5^, ^6^, ^7^, ^8^ ^]^ temperature gradients^[^ ^9^, ^10^, ^11^, ^12^ ^]^ or via light stimulation,^[^ ^13^, ^14^ ^]^ and many more. Such modifications are not only limited to planar interfaces, but can be extended to particles of various sizes ranging from a few nanometers^[^ ^15^, ^16^, ^17^, ^18^ ^]^ up to hundreds of microns.^[^ ^19^, ^20^, ^21^, ^22^ ^]^ Especially on the micrometer scale the separation of such surface‐coated particles from those “less, unreacted, or uncoated” particles is still challenging, as there is a lack of separation techniques that only target the interfacial properties. Besides common examples to fraction microparticles by size differences,^[^ ^23^, ^24^, ^25^, ^26^, ^27^, ^28^, ^29^ ^]^ material sensitive separation methods are to date barely developed and mostly require special material properties of the particles, for example, magnetic properties,^[^ ^30^, ^31^, ^32^ ^]^ or differences in polarization.^[^ ^33^, ^34^, ^35^ ^]^ However, magnetic or dielectric properties located at the interface will have a weak impact in comparison to the particle bulk material. Therefore, for particles with subtle differences in interfacial morphology, the separation with classical and established material‐sensitive separation techniques is inadequate and new approaches are required.

Such a technique has been demonstrated recently using light‐triggered chemical phoresis/osmosis in combination with microfluidic technology, whereby the separation of particles by differences in the surface morphology is achieved through variation of retention along a pressure‐driven fluid flow.^[^ ^36^ ^]^ The key to the interfacial sensitivity lies in a photo‐sensitive azobenzene‐containing surfactant, which simultaneously stabilizes the microparticles and provides a phoretic/osmotic activity under light illumination with appropriate wavelengths.^[^ ^37^ ^]^ This effect can hover microparticles into higher planes within the channel, where the strength and extend of the hovering height depends on the interfacial properties of the microparticles.^[^ ^36^ ^]^ Then particles experience an increased shear^[^ ^38^, ^39^, ^40^ ^]^ proportional to the light‐induced hovering height, and can be separated through differences in the translation velocity across a rectangular microfluidic channel.^[^ ^36^ ^]^

The phoretic/osmotic activity results from the *local*‐light‐driven diffusioosmosis (*l*‐LDDO), that is, a light‐induced chemical gradient of surfactant *cis* isomers in the vicinity of the microparticles based on differences in photoisomerization rate in the bulk solution and on the interface. In this way hard and soft microparticles or random micron‐sized objects become a source of a laterally inhomogeneous excess of *cis*‐isomers that diffuses away from the particles. This leads to an effective concentration gradient and a corresponding generation of the diffusioosmotic flow, a kind of interfacial fluid flow.^[^ ^41^, ^42^ ^]^ Furthermore, due to the symmetry break from sedimented particles located at the planar interface and the remotely activated “phoretic activity” induced by the *l*‐LDDO,^[^ ^43^, ^44^ ^]^ this results in a vertical displacement of sedimented particles from an interface into higher plane,^[^ ^36^ ^]^ similar to phoretically active colloids near walls that have been theoretically modeled.^[^ ^40^, ^45^ ^]^

To separate equally sized microparticles that differ in interfacial morphology, significant differences in phoretic activity are required, for example, porous versus plain microparticles.^[^ ^36^ ^]^ However, we expect/assume that the majority of microparticles varying in their interfacial morphology presumably will have weak phoretic properties (interfacial morphology will not always be porous). To increase the versatility with respect to various particle types only varying in interfacial surface coating, one needs to enhance the phoretic/osomotic activity of the microparticles. In this article, we demonstrate that this weak phoretic activity can be amplified by additional heating of the bottom interface where microparticles are sedimented. The combination of bottom heating and light enhances the *l*‐LDDO strength for differently grafted microparticles. This is demonstrated for polymer grafted versus uncoated plain silica microparticles, which possess a natural weak phoretic/osomotic activity.^[^ ^36^ ^]^ Thus, we expect that varying polymer grafts on microparticles presumably will have similar phoretic activity. However, polymer grafting represents one of the most attractive tools to functionalize the particle interface. By separating particles grafted with different polymer brushes, we aim to demonstrate the scope of light‐driven diffusioosmosis.

There are various methodologies to create brushes via reversible deactivation radical polymerization techniques,^[^ ^46^, ^47^ ^]^ including surface initiated (SI) methods based on atom transfer radical polymerization,^[^ ^48^ ^]^ as well as strategies based on reversible addition‐fragmentation chain‐transfer (RAFT) polymerization.^[^ ^49^ ^]^ For the latter, a differentiation between the R‐group approach^[^ ^50^ ^]^ (chains growing on the surface) and the Z‐group approach^[^ ^51^ ^]^ (chains growing in solution and deactivating on the surface) is necessary.

It combines ease in preparation with a reliable control over the grafted polymer chains.

Photo‐initiated methods like photo‐electron transfer (PET) RAFT polymerization^[^ ^52^ ^]^ or photo‐iniferter (PI)‐RAFT polymerization^[^ ^53^ ^]^ offer additional advantages like spatial control^[^ ^54^ ^]^ or oxygen tolerance.^[^ ^55^ ^]^ In PI‐RAFT polymerization the direct activation of the chain‐transfer agent (CTA) by light, resulting in a direct generation of radicals on the interface, leads to high levels of control, and the use of shuttle CTAs enables monitoring of polymer length of grafted brushes.^[^ ^56^ ^]^

Using this strategy, we aim to create grafted silica microspheres with polymers that only differ slightly in their polarity, and demonstrate how the combination of two double complementary non‐invasive stimuli (temperature and light) enables the separation of particles from each other.

Further, triggering the volume phase transition temperature (VPTT) of one of the polymers changes its interfacial properties and enables a potential separation via the phoretic/osmotic vertical displacement of particles.

## 2 Results and Discussion

### 2.1 Synthesis

To investigate the fractioning of equally sized microparticles featuring varying surface morphology, we grafted silica microparticles with polymer brushes based on poly(*N*‐isopropylacrylamide) (pNIPAM) or poly *N*‐acryloyl morpholine (pNAM) (further abbreviated as pNIPAM@SiO_2_ and pNAM@SiO_2_). Both polymers possess a VPTT in water. However, while pNIPAM responds to temperatures around 32 °C,^[^ ^57^ ^]^ the more polar pNAM is reported to have cloud points around 88 °C.^[^ ^58^ ^]^

Amino‐functional silica microspheres were first made to react with the NHS ester of 2‐[(ethoxythioxomethyl)thio]‐2‐methylpropanoate (MeXan). The CTA was activated via UV light (365 nm) to initiate the rapid polymerization of NAM or NIPAM. Using a shuttle CTA (free CTA polymerizing simultaneously in solution)^[^ ^50^, ^59^ ^]^ the molecular mass of polymer grafts can be deduced resulting in *M*_n_ values above 20 000 g mol^−1^ and high dispersities. The latter was suspected due to the limited chain transfer capabilities of MeXan with monomers used. However, a high dispersity in polymer brushes does not interfere negatively with functionality.^[^ ^55^ ^]^ To gather more evidence of successful grafting thermogravimetric analysis (TGA) was conducted (Figure S4, Supporting Information), and based on weight loss the grafting density was estimated to be *σ* ≈ 0.3 chains nm^−2^ (pNIPAM@SiO_2_) and *σ* ≈ 0.7 chains nm^−2^ (pNAM@SiO_2_). Moreover, the grafting protocol was also performed on silica nanoparticles to follow the process via light scattering. A more detailed description can also be found in the Supporting Information (see Section S1 and S2).

### 2.2 Separation

**Figure 1:**
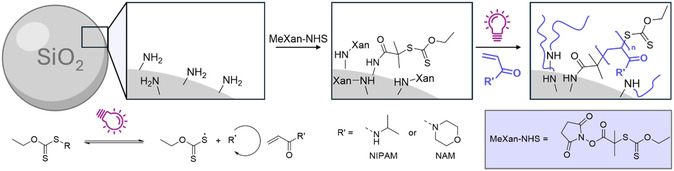
Schematic representation of polymer brush formation via surface‐initiated PI‐RAFT polymerization from silica microspheres. Amino functional particles were functionalized with MeXan and polymers were grown in a grafting from fashion under UV light.

**Figure 2:**
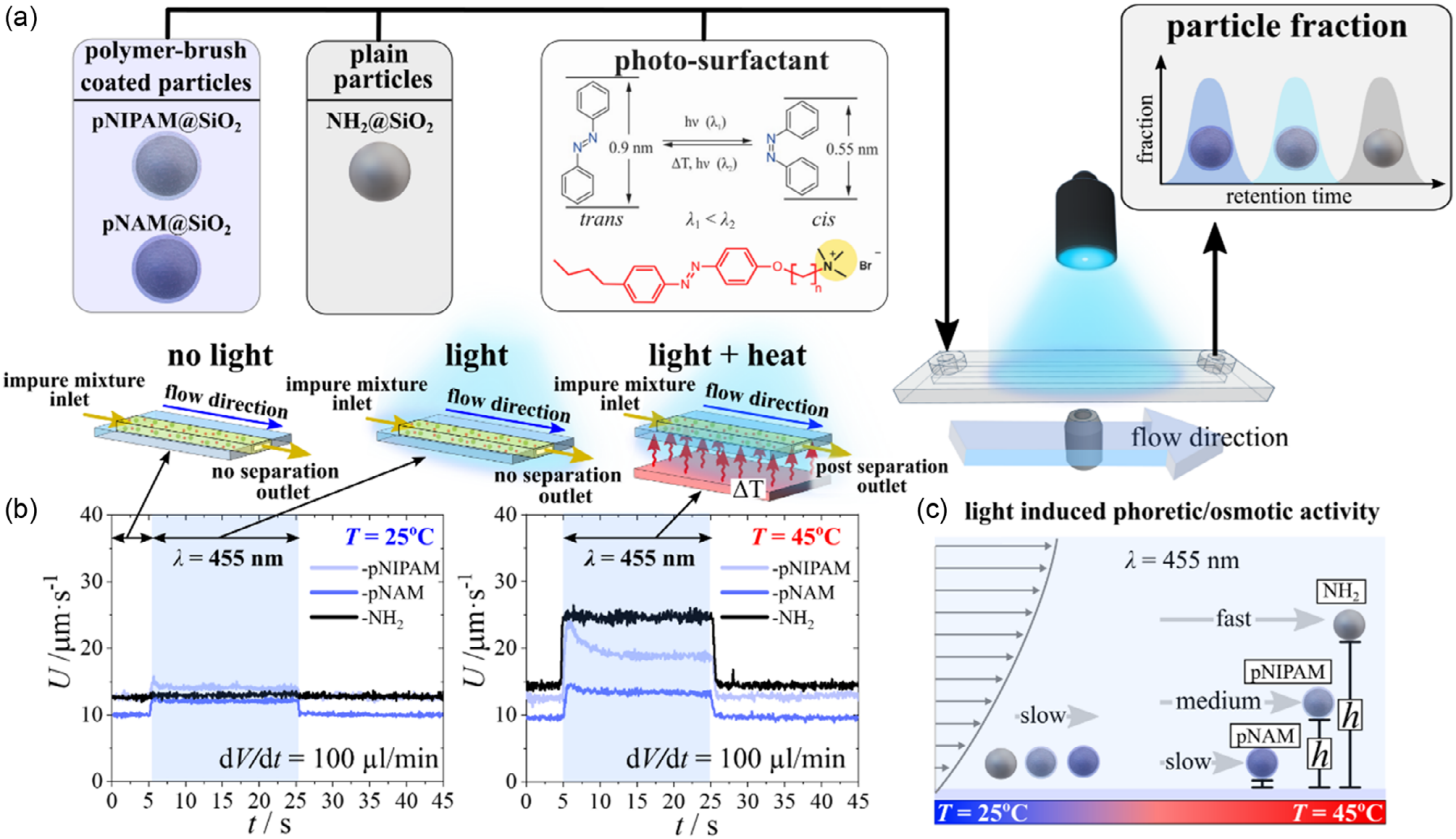
Schematic representation of the experimental setup. a) Microparticles (*D* = 4 μm) coated with pNIPAM or pNAM polymer brush, or amino functionalized are mixed with an aqueous solution of an azobenzene surfactant and injected into a microfluidic channel with rectangular geometry. The bottom of the channel can be heated and simultaneously illuminated with blue light. b) Average velocity as a function of time at 25 and 45 °C. Data extracted from Video S2 and Video S3 (Supporting Information). c) Schematic representation of the velocity drift motion gain. The osmotic activity of the particles hovers them from the bottom interface. The stronger shear gradient provides a different drift motion, which potentially yield into a collection at different times of retention. The average sample size of the particles per frame b) at *T* = 25 °C (left) is 1833 ± 72 (NH_2_@SiO_2_), 5442 ± 82 (pNAM@SiO_2_), 2390 ± 19 (pNIPAM@SiO_2_) and *T* = 45 °C (right) is 807 ± 34 (NH_2_@SiO_2_), 6749 ± 33 (pNAM@SiO_2_), 2950 ± 85 (pNIPAM@SiO_2_). Sample size collected from tracking a 4 k resolution image.

To investigate the fractioning of equally sized functional particles but with varying difference in their surface morphology we mixed silica microparticles (diameter (*D* = 3.9 ± 0.2) μm) with either primary amines (–NH_2_), pNAM grafts, or pNIPAM grafts (see **Figure** **1**) with an aqueous solution containing a photosensitive azo‐benzene surfactant (*c* = 2 mM, **Figure** **2**a). We injected the dispersion (≈minimum 1 day equilibration time) into the rectangular microfluidic chamber with following channel dimensions: *h* = 0.54 mm, *d* = 3.8 mm, *L* = 2.4 cm. After the microparticles sediment on the bottom glass wall, and under pressure driven fluid flow (syringe pump), particles follow the translational drift motion:^[^ ^60^ ^]^

(1) $$U=\frac{1}{2}\;a\;s$$

proportional to the particle radius *a* and applied shear stress *s* of unperturbed flow in the center of the particle.^[^ ^36^, ^60^ ^]^ Data in Video S2 (Supporting Information) for NH_2_@SiO_2_, pNIPAM@SiO_2_, and pNAM@SiO_2_ under flow with a flow rate of 100 μL min^−1^ and summarized in Figure 2b exhibit that all particles have similar drift motion when no light is applied. However, as soon as samples are illuminated with blue light (*λ* = 455 nm), all particle types experience an increased drift motion during the illumination period (illustrated with blue rectangular), which dissipates back to initial velocity when illumination is ceased. The light‐induced drift motion (LIDM) results from increased shear stress experienced by particles as they hover above the bottom interface of light‐induced phoretic/osmotic activity. The latter is proportional to the *l*‐LDDO,^[^ ^36^ ^]^ caused from the dynamic exchange of between *trans* and *cis* isomers with different extends for plain and polymer brush grafted interfaces.^[^ ^42^ ^]^ Data exhibits small differences between all different particles types, for instance amino (NH_2_@SiO_2_) versus polymer‐coated particles (pNIPAM@SiO_2_, pNAM@SiO_2_). When considering the separation of microparticles, one needs to aim for a strong difference in the translation velocity^[^ ^61^ ^]^

(2) $$\Delta U=\left|U_{1}–U_{2}\right|$$

with Δ*U* as the velocity difference from *U*_1_ and *U*_2_ of particle type 1 and 2. Thus, under conditions as measured in Video S2 (Supporting Information) and Figure 2b, particles cannot be separated via different retention times along the elongated rectangular channel. To increase the motion contrast Δ*U*, we heated samples to 45 °C from the bottom of the microfluidic channel, as illustrated on top of Figure 2b. Further we applied the same experimental conditions with a flow of cold aqueous solution of photosensitive surfactant solution, *T* = 22 °C, (syringe reservoir is intentionally not heated) at a flow rate of 100 μL min^−1^. We observe under light illumination (*t* = 5–25 s) in Video S3 (Supporting Information) and summarized in Figure 2b, a pronounced difference of LIDM between NH_2_@SiO_2_, pNIPAM@SiO_2_, and pNAM@SiO_2_ microparticles.

Apparently complementary stimulus via (I) bottom heating and (II) light illumination is a simple method to drastically increase the value of Δ*U* of differently functionalized particles, and significantly increase the fractionation of microparticles via different retention times.

**Figure 3:**
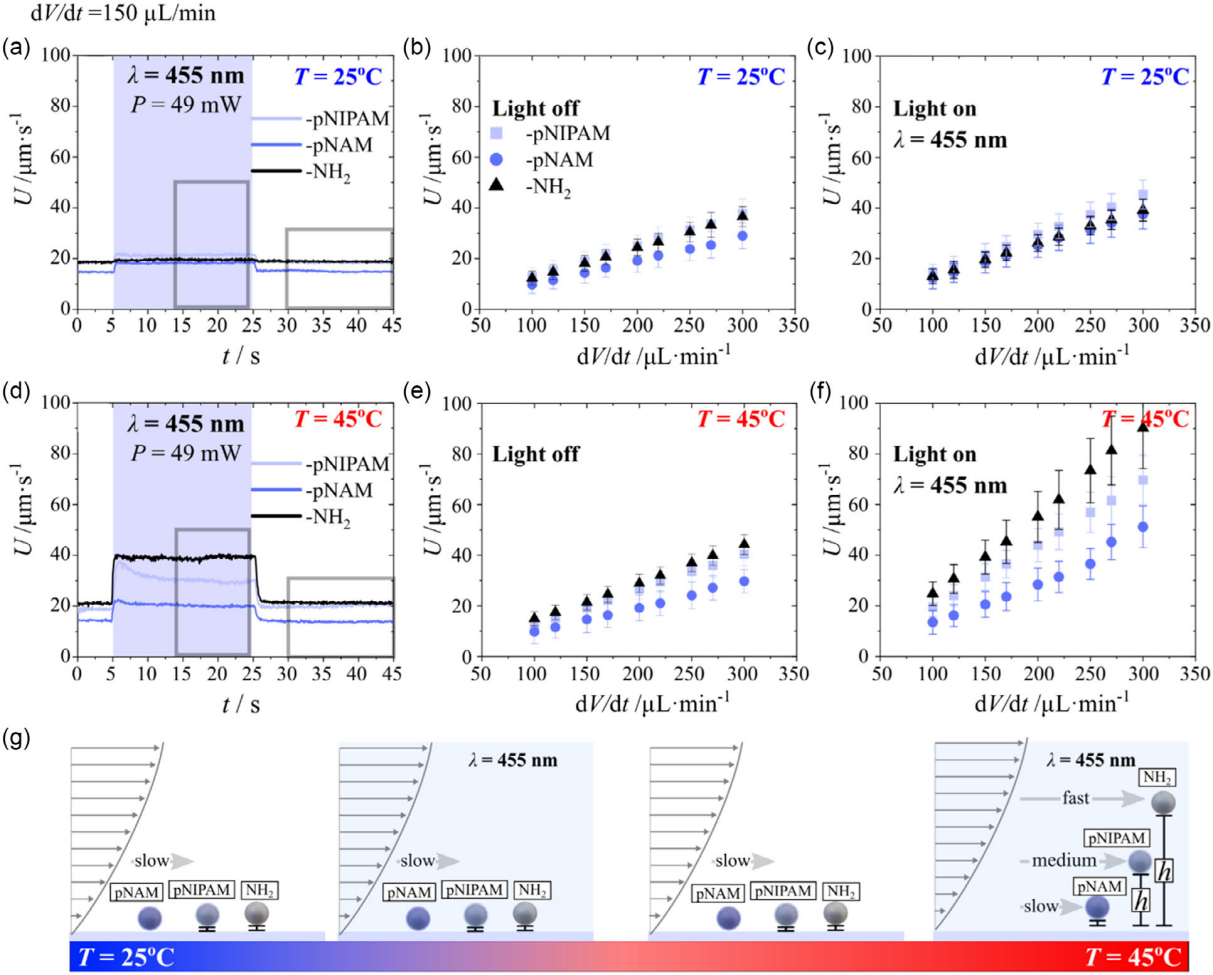
a,d) Average particle velocity as a function of time at a flow rate of 150 μL min^−1^ for a temperature at a) 25 °C and d) 45 °C, blue rectangle illustrates the illumination time frame (*λ* = 455 nm). Data extracted from Video S4 and Video S5 (Supporting Information). Grey rectangle illustrates the average velocity displayed as a function of the flow rate in (b,c and e,f). g) Schematic illustration of the correlation between translational velocity of the particle and particle height without and with light illumination (*λ* = 455 nm). The average sample size of the particle per frame for data in a) is 1611 ± 25 (NH_2_@SiO_2_), 6002 ± 98 (pNAM@SiO_2_), 2310 ± 30 (pNIPAM@SiO_2_) and d) is 1022 ± 94 (NH_2_@SiO_2_), 6483 ± 34 (pNAM@SiO_2_), 2631 ± 35 (pNIPAM@SiO_2_). Sample size collected from tracking a 4 k resolution image. For data displayed in (a),(d), (c) and (f) corresponding hovering height is calculated displayed in Figure S7 (Supporting Information).

We repeated the experiment under the same experimental conditions without and with heating up to *T* = 45 °C with a different flow rate while keeping all other parameters fixed. An example, time resolved motion profile is shown for a flow rate of 150 μL min^−1^ and illumination power of 49 mW (455 nm) for all used microparticles, is shown in Video S4 (Supporting Information) (*T* = 25 °C) and Video S5 (Supporting Information) (*T* = 45 °C), and summarized in **Figure** **3**a,d. Further we plotted the average velocity in steady state conditions, illustrated from the grey rectangle in Figure 3a,d, with and without illumination, displayed in Figure 3b,c,e,f. Experimental data exhibits a linear relation between the value of *U* and the applied flow rate, in good agreement with expected laminar fluid flow regime.^[^ ^39^ ^]^ Experimental data obtained at room temperature demonstrates that the velocity difference, Δ*U*, between all particles with and without illumination is insufficient for fractionation at all flow rates (Figure 3b,c). At temperature for *T* = 45 °C the experimental data exhibits a similar trend without illumination, but under illumination the value of Δ*U* increases between all particles and increases with increasing flow rate (Figure 3e,f).

Only the complementary double stimulus via heating and light illumination (for principal mechanism and detailed discussion see Section 2.3) yields a pronounced difference in the velocities, with increasing impact toward higher flow rates. This is because the light illumination induces the *l*‐LDDO effect in close vicinity of the microparticles, resulting in phoretic/osmotic chemical activity of the microparticles induced from the photosensitive surfactant. This can hover the sedimented microparticles, apparently with strong variances in hovering height between NH_2_@SiO_2_ > pNIPAM@SiO_2_ > pNAM@SiO_2_. We calculated the height from the center point of the micro particle relative to the bottom of the interface^[^ ^36^ ^]^ displayed in Figure S7 (see details Supporting Information, Section S3). Data exhibits that the value of *h* varies between 1.95 and 2.00 μm when the phoretic activity is low (light plus room temperature) or not activated (light off). This is in good agreement with theoretical expectations^[^ ^36^ ^]^ as values of *h* are close or equal to the size of the particle radius *a* = 1.95 μm = 3.9/2 μm. This means that the particles are basically sedimented, *h*–*a* ≈ 0 μm. But as soon as the double stimulus is switched on (light + heat), values of *h* increases above the particle radius with height variances $h_{{\text{NH}}_{2}}$ ≈ 2.87–3.27 μm > *h*_pNIPAM_ ≈ 2.44–2.69 μm > *h*_pNAM_ ≈ 2.00–2.22 μm. We observe that NH_2_@SiO_2_ particles show the highest lift‐off tendency from the interface, up to 1.27 μm, while pNIPAM@SiO_2_ and pNAM@SiO_2_ particles lift up to 0.69 and 0.22 μm. Generally, the faster the flow, the stronger the average elevation of the microparticles (see Figure S7d, Supporting Information). This may result from a complex interaction of two acting lift forces acting with the bottom glass interface, (I) the phoretic activity of the micro particle and (II) the wall lift force. For (II) the impact of the wall lift force increases with increasing flow rate, due to a gradual change from conventional microfluidics at low flow rates (Stokes flow region with very low Reynolds number, Re ≪ 1,) into more inertial microfluidics (≈1 < Re < ≈ 100) at higher flow rate,^[^ ^62^, ^63^ ^]^ in reasonable agreement with data in Figure S7d (Supporting Information). From the experimental data in Figure 2b, Figure 3 it can be interpreted that the sensitivity toward the very slight difference in interfacial properties of microparticles can only be achieved by applying a complementary stimulus. In the following we seek to explain the experimental observations, where heating (only from the bottom interface) simultaneously changes conditions near the planar interface of the channel, as well as the interfacial properties of pNIPAM@SiO_2_ microparticles in comparison to pNAM@SiO_2_ and NH_2_@SiO_2_. The light illumination activates the chemical activity, and combination of both changes the strength of *l*‐LDDO between the three particle types ‐ the necessary key to superior interfacial sensitive fractioning of microparticles.

### 2.3 Separation Mechanism

Polymers,^[^ ^64^, ^65^ ^]^ microgels,^[^ ^66^ ^]^ and polymer brushes^[^ ^67^ ^]^ are in complex dynamic interaction with surfactants, which can effectively penetrate the polymer matrix.^[^ ^68^, ^69^ ^]^ The interactions are not solely dominated by electrostatics but also 50% the of accumulated surfactants are dominated by hydrophobic interactions.^[^ ^68^ ^]^ This overall interaction usually leads to an overcharging of polymer networks (microgels)^[^ ^66^, ^67^ ^]^ or of particles with polyacrylic acid (pAA) brushes once a certain surfactant concentration threshold is exceeded.^[^ ^70^ ^]^ The mentioned examples are anionic polymer networks. Thus, we expect a similar overload from surfactant molecules for pNIPAM and pNAM polymer brushes, as indicated from strong positive zeta potential values without illumination and under blue light illumination in surfactant solution, compared to neutral values in Millipore water (Figure S5, Supporting Information). This is also why under illumination the more hydrophilic *cis* isomer becomes expelled from the polymer brush matrix,^[^ ^66^, ^71^, ^72^ ^]^ making polymer brush‐coated particles phoretic/osmotic active, too.^[^ ^70^ ^]^ However, in comparison to pAA, pNAM, and pNIPAM, non‐charged brushes lead to an expectedly weaker complexation tendency and thus a weaker phoretic activity for polymer‐coated particles, in comparison amino‐coated particles.

Further we assume that both particle types grafted with pNAM and pNIPAM have a similar complexation tendency with the cationic photosensitive surfactant (≈*trans* isomer) below their VPTT. Under illumination with blue light (455 nm) the phoretic activity will be similar, leading to a similar light‐induced drift motion from phoretic hovering. This means that a fractioning between both particles along elongated channels will be impossible due to almost no differences without illumination (no activated phoretic activity, Figure 2b, Figure 3b,e) or with illumination at room temperature (activated phoretic activity, Figure 3c), in reasonable agreement with experimental data.

Irrespective of the complex interaction of phoretic activity and corresponding hovering tendency, the microparticles exhibit a pronounced translational motion difference only if a double stimulus, heat and light illumination, is applied. This means that the sensitivity, with respect to particle fractionation in only slight differences in interfacial morphology, is drastically enhanced at higher temperatures and under light illumination of appropriate wavelength, with greater impact toward higher flow rate (see Figure 3f).

The hovering of particles can be theoretically described through phoretic activity, as described in our previous publication.^[^ ^36^ ^]^ This complex interaction effectively yields to a higher focal plane, where a particle experiences the stronger shear stress as a function of the hovering height and particle radius (see Figure S7, Supporting Information). Data in Figure 2b and 3b,c exhibits that the phoretic activity of the particles at a temperature of 25 °C is weak and so the LIDM is close to that without illumination, in good agreement with previous literature for plain silica microparticles.^[^ ^36^ ^]^

**Figure 4:**
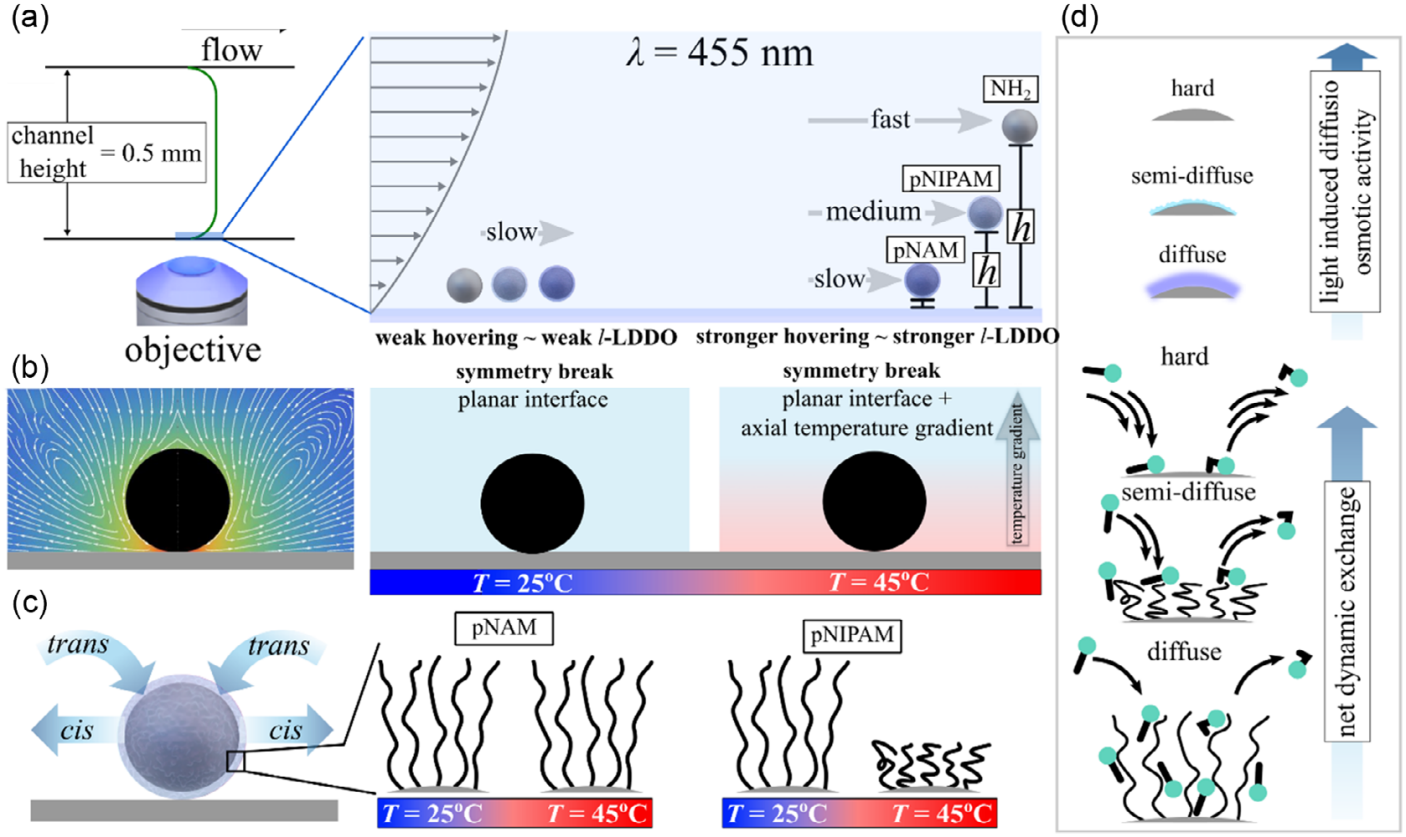
Sketch of principle mechanism of the separation of differently functionalized microparticles. a) Particles located at the bottom of the planar interface will be hovered from the phoretic activity and experience a different shear stress as a result of the height. Particles are stronger hovered when heating up the channel from the bottom interface. b) Illustrated fluid field from *l*‐LDDO. The symmetric break results in a hovering of the particle. The overall symmetry break is amplified by temperature gradient, facing from the bottom toward the bulk solution. c) The *l*‐LDDO is proportional to the exchange dynamics of *trans* and *cis* isomers. Rectangles illustrate at molecular scale the polymer brush structure at temperature *T* = 25 °C and *T* = 45.°C for pNAM and pNIPAM. d) Illustrated dynamic exchange kinetics for hard, semi‐diffuse, and diffuse interfacial structure and illustrated hovering tendency.^[^ ^36^ ^]^

We interpret the two major interactions influencing the increased strength of the phoretic activity of the particles and deviations between particle types as a function of the temperature, which increases the sensitivity with respect to the LIDM. The first interaction is the global heating of the microfluidic channel from the bottom interface as illustrated in **Figure** **4**b. It causes the heat to dissipate toward the bulk solution and induces a temperature gradient, facing from the interface upwards. The heat dissipation can be in the range of several micrometers and is therefore on the same size scale as that of microparticles and light‐induced hovering height.^[^ ^36^ ^]^ We hypothesize that this gradient provides an additional symmetry break as illustrated in Figure 4b, and amplifies the asymmetric fluid flow profile close at the interface. Thus, the LIDM gain is stronger in comparison to experiments at room temperature, where no temperature gradients can be expected, in reasonable agreement with experimental data displayed in Figure 2b and 3. Apparently, slight changes in the interfacial morphology have a great impact and are the second major interaction enhancing the sensitivity of the fractioning of the particle types. Heating the sample above the VPTT of pNIPAM ≈ 32 °C and below the VPTT of pNAM ≈ 88 °C results in a collapse of pNIPAM brushes, while the pNAM brushes remain swollen. In the first approximation the interfacial properties of the particles change from a more diffuse/soft into a semi‐diffuse/less soft interface. This may influence the net dynamic exchange of both surfactant isomers and influences the light induced *cis* isomer gradient. In general, experimental data exhibits a gradual decaying strength of LIDM from hard (–NH_2_) > semi‐diffuse (collapsed pNIPAM) > diffuse (pNAM) interfacial morphology at 45 °C, presumably due to a reduction in light‐induced exchange dynamics between *trans* and *cis* isomers. For pAA brushes grafted on the same set of silica particles at similar surfactant concentrations (≈2 mM), we demonstrated that despite a pronounced *trans*‐isomer accumulation tendency,^[^ ^70^ ^]^ 1) the exchange kinetics^[^ ^42^ ^]^ are reduced from a lower photo‐isomerization kinetics of surfactants complexed with polymers^[^ ^65^ ^]^ in comparison to bulk solution,^[^ ^73^ ^]^ 2) from lower *trans*‐isomer adsorption constant for polymeric networks^[^ ^71^ ^]^ in comparison to silica interfaces^[^ ^74^ ^]^ and 3) reduced diffusivity of the surfactant isomers inside the polymer matrix.^[^ ^70^ ^]^ All three effects (1–3) generally lower the chemical activity of the polymer‐grafted particles (pNIPAM@SiO_2_ and pNAM@SiO_2_ among hard microparticles (NH_2_@SiO_2_). However, the collapsed pNIPAM at 45 °C is a “more semi‐diffuse” interface, resulting in a net chemical activity in between hard and diffuse interface (Figure 4d). Therefore, we observe in Figure 2b and 3f a gradual decaying strength of LIDM (≈hovering height, ≈chemical activity, see Figure 4a,d) from hard (–NH_2_) > semi‐diffuse (collapsed pNIPAM) > diffuse (pNAM) interfacial properties of particles at *T* = 45 °C.

**Figure 5:**
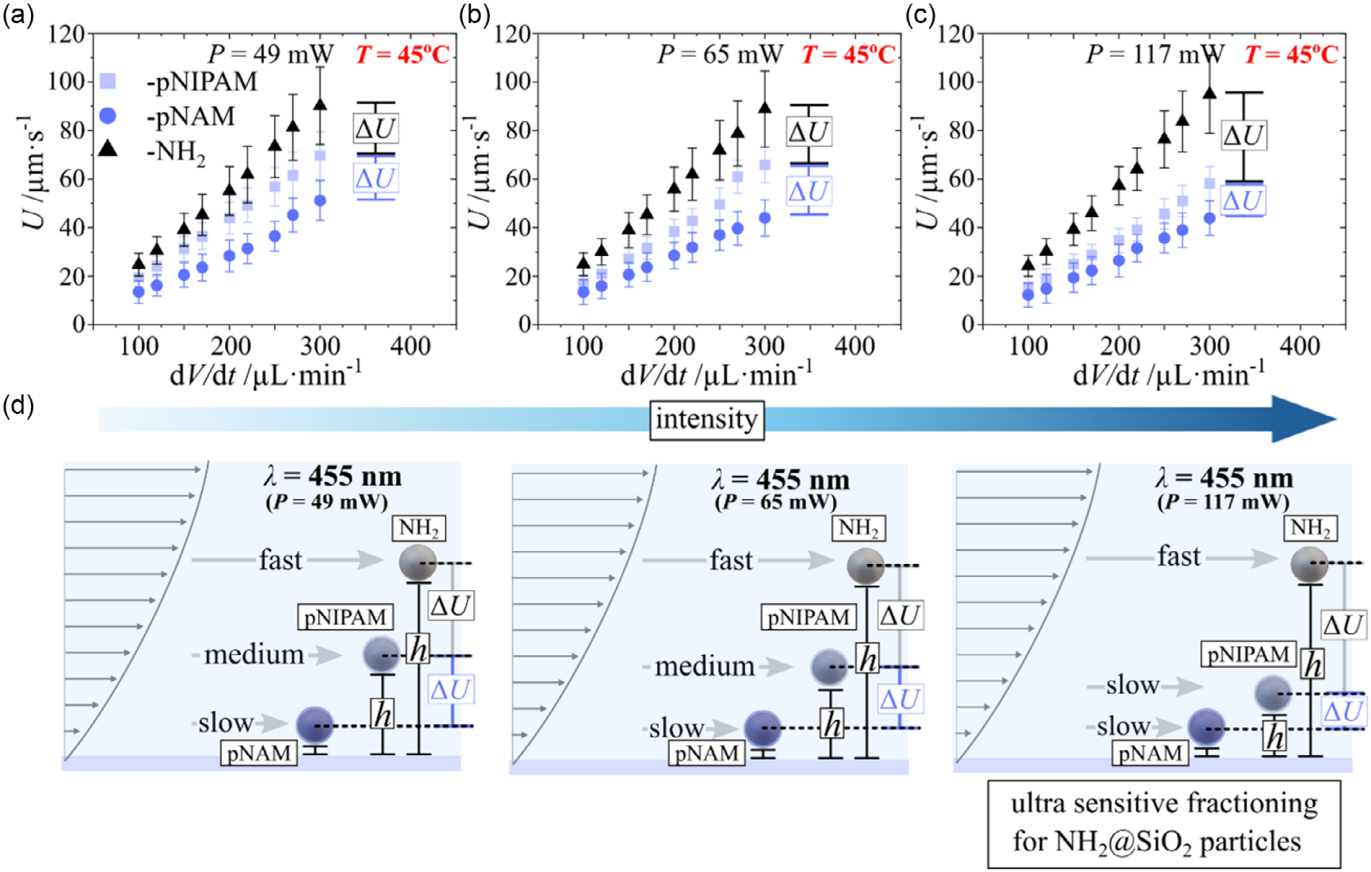
a–c) Light‐induced average velocity as a function of applied flow rate at 45 °C classified by the applied light power: a) *P* = 49 mW, b) *P* = 65 mW, c) *P* = 117 mW, d) cartoon of hovering height and motion difference for the three different particle types. Corresponding hovering height is calculated in Figure S8 (Supporting Information).

Further we investigated the LIDM as a function of applied light intensity, displayed in **Figure** **5**, and calculated the hovering height in Figure S8 (Supporting Information). We observe a gradual reduction of the motion difference, Δ*U*, as applied intensity between pNAM and PNIPAM microparticles, while simultaneously increasing between pNIPAM and amino‐coated microparticles (–NH_2_) (see Figure 5a–c). Apparently data indicates for pNAM@SiO_2_ and NH_2_@SiO_2_ microparticles that the dynamic exchange is already in the adsorption limited regime,^[^ ^71^ ^]^ and increasing intensity does not have a significant impact on the phoretic/osmotic activity (≈hovering height). The generally weaker activity of the pNAM‐coated interface versus NH_2_‐coated interface relies on the swollen polymer brush characteristics (≈diffuse interface). The deeply penetrated surfactant inside the swollen pNAM polymer brush has a longer diffusion path length and therefore a longer diffusion time in comparison to the hard interface of NH_2_@SiO_2_ particles. The light‐induced dynamic isomerization between *trans* and *cis* isomers, proportional to the applied light intensity,^[^ ^71^, ^73^ ^]^ influences the average lifetime of one isomer, with decreasing time as intensity increases. Then an increase in the intensity increases the oscillation frequency between more hydrophobic *trans* isomers (strong accumulation tendency in polymer brush) versus the more water‐soluble *cis* isomers (weak accumulation in polymer brush), and surfactant isomers remotely accumulate and release from brush interior proportional to the switching frequency.^[^ ^70^ ^]^ Too high switching frequencies yields an effective locking of both isomers in the polymer matrix and therefore reduces net dynamic exchange.^[^ ^70^ ^]^ Since the diffusivity of the surfactant inside a polymer brush can be expected to be lower, presumably diffuse polymer interfaces in general will have a weaker light induced exchange and a weaker phoretic activity in comparison to hard interfaces. We expect that neutral polymer brushes pNAM and pNIPAM to accumulate less surfactants in comparison to pAA brushes, those *l*‐LDDO strength measured to be around three times stronger in comparison to plain silica particles.^[^ ^70^ ^]^ Thus pNIPAM@SiO_2_ and pNAM@SiO_2_ particles have a weaker phoretic activity (≈LIDM) in comparison to NH_2_@SiO_2_. We would like to highlight that applied light power is in the range of 65–117 mW for an illumination area of 0.25 cm^2^ and corresponding intensities are in the range of *I* ≈ 260–460 mW cm^−2^, far away from the critical light intensity to enter the adsorption limited regime around 4 mW cm^−2^ for pMAA@SiO_2_ particles.^[^ ^70^ ^]^

In contrast, the collapsed pNIPAM interface, representative of a semi‐diffuse interface model, enters the transition regime between hard and diffuse interfaces. We hypothesize that the locking tendency at higher switching frequency (≈higher intensities) may have a strong impact on the effective net exchange due to neither being a hard nor a completely diffuse interface. Then the critical photoisomerization frequency, where strong net diffusion limitations for the entire collapsed brush in vertical displacement with respect to particle´s interface, is in the applied intensity regime between those to hard (≈NH_2_@SiO_2_) and soft (≈pNAM@SiO_2_) interfaces. Thus, applied light intensity drastically enhances diffusion limited regime for pNIPAM@SiO_2_ particles and reduces net exchange potential and therefore the phoretic activity and LIDM.

Irrespective of the complex interaction, increasing light intensity increases the sensitivity in fractioning potential between amino (–NH_2_) versus the polymer‐coated microparticles, interpreted from the increasing consecutive value of Δ*U* for all flow rates. The demonstrated example illustrates a potential smart and simple tool to increase the sensitivity with respect to fractioning a selective particle type.

### 2.4 Practical Example of Separation

To demonstrate the separation via differences in velocity we used the developed method in a proof‐of‐concept experiment. In practice, for successful separation of microparticles at different locations the superposition of initially and finally located particles plays an important role. As discussed in previous literature, separation may be achieved via different crossing times along the rectangular channel,^[^ ^36^ ^]^ however, a more convenient strategy is reported elsewhere.^[^ ^61^ ^]^ This approach suggests cumulative operation cycles (separation cycles) in (I) forward flow direction under light illumination (phoretic activity of colloids switched on) and (II) reverse flow direction without light illumination (phoretic activity of colloids deactivated). With the combination of both (I) and (II) a gradual superposition change of a particle fraction can be achieved, as long as differences in motion Δ*U* > 0 during light illumination (≈forward flow) are present.

**Figure 6:**
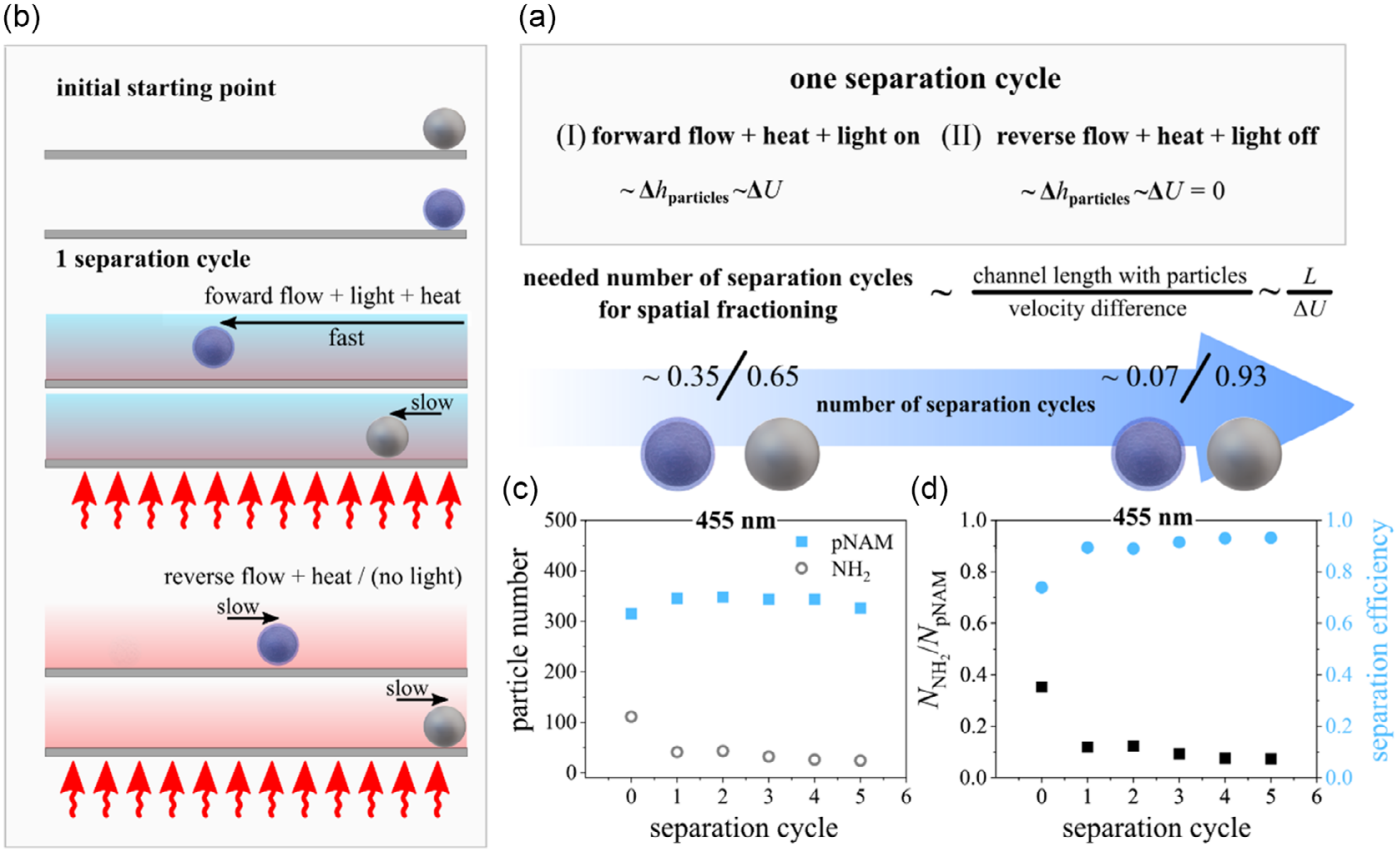
Schematic illustration of an effective spatial fractioning inside a rectangular microfluidic channel: a) Illustration of one separation cycle with first (I) forward flow in combination with light illumination and then subsequent second (II) reverse flow without illumination. The combination of (I) and (II) is one separation cycle. The number of separation cycles depends on the channel length with deposited particle and the velocity difference under light illumination, Δ*U*. b) Illustration of spatial particle mixture separation for one operation cycle. The principle is demonstrated with a binary mixture of NH_2_@SiO_2_ and pNAM@SiO_2_ silica microparticles measured from snapshot series (Figure S13, Supporting Information) and Video S6–S11 (Supporting Information) recorded area 1000 μm^2^ = 100 · 100 μm^2^) near the channel inlet. c) Local particle number and d) particle ratio (dark points) of NH_2_@SiO_2_ to pNAM@SiO_2_ per separation cycle. Displayed separation efficiency (blue points) per separation cycle. For details see Supporting Information Section S4.

With the reverse flow “non” or “less” phoretic active particles effectively remain at the same position/area, while “strong” phoretic active particles gradually change the superposition until disappearing from the area or microfluidic channel.^[^ ^61^ ^]^ Then the number of separation cycles *n* is proportional to the ratio of distance of covered particles inside the channel *L* and light‐ induced velocity difference Δ*U* for the particle mixture.^[^ ^61^ ^]^ This strategy of oscillating flow directions (**Figure** **6**a,b) not only allows a spatial fractioning at the desired area, but also minimizes the required channel length as a chromatographic approach to separate particles.

We used this principle while additionally providing heat from the bottom of the surface. This causes the weak phoretic active particles to possess a stronger phoretic activity and thus a stronger motion difference Δ*U* under light illumination (≈forward flow, Figure 6), while in reverse flow the value of Δ*U* is ≈0 (for same sized particles). This principle of particle fractioning under double stimulus is demonstrated for a mixture of NH_2_@SiO_2_ and pNAM@SiO_2_ particles. The illumination power is set to *P* = 117 mW, because in such conditions the value of Δ*U* between pNAM@SiO_2_ and NH_2_@SiO_2_ is maximized (see Figure 5). Thus, we expect the lowest amount of separation cycles under such conditions. To distinguish between NH_2_@SiO_2_ and pNAM@SiO_2_ in the experimental setup we use a surfactant‐dye complex with fluorescein,^[^ ^75^, ^76^ ^]^ although the emission of NH_2_@SiO_2_ relative to bulk solution emission intensity is very low. According to the more positive interface from grafted amino moieties, more negatively charged fluorescein accumulates at NH_2_@SiO_2_ in comparison to the neutral pNAM@SiO_2_, but the overall emission quality of the particles is poor. Image acquisition has been collected near microfluidic channel inlet (see details Supporting Information Section S4) to exclude more incoming microparticles in the investigated area.

We quantified the ratio of NH_2_@SiO_2_ and pNAM@SiO_2_ by counting the remaining particles from the combination of bright‐field and emission microscopy after each separation cycle, displayed in Video S6–S11 (Supporting Information) (one video corresponds to one separation cycle). The data in Figure 6c,e exhibits that the fraction of NH_2_@SiO_2_ particles (0.26 = 111/315) decreases with increasing separation cycle and almost vanishes after the 5th cycle (0.07 = 24/350). In total we achieved spatial fractioning for the investigated area (1000 μm^2^ ≈ 100 · 100 μm) in a time frame of around 450 s (5 · 90 s = 5 cycles), which demonstrates a relatively fast and feasible way to separate weakly phoretically active microparticles. This interpretation is additionally supported by calculating the separation efficiency (see details in Supporting Information, Section S4.4).^[^ ^77^ ^]^ Data in Figure 6d exhibits that with an increasing separation cycle the separation efficiency increases up to 93%. This demonstrated example illustrates that even weakly phoretically active particles can be accurately separated, although more separation cycles are needed. We interpret this by comparing the actual separation efficiency ≈93% (Figure 6d, last cycle) with the literature for a mixture of strong and weakly active microparticles to be 97%, where the value of Δ*U* is higher in comparison to data shown in Figure 6.^[^ ^61^ ^]^

This method usually requires particles sedimented at the glass–bottom interface. This means a precise fractioning is only possible if sedimented particles do not aggregate in multilayer formation, due to increased velocity in higher layers. Thus, according to our empirical observations, the concentration of microparticles should not exceed a value of 3 mg mL^−1^ (bulk concentration) since above this the local concentration of sedimented microparticles at the glass‐water interface is too high.^[^ ^78^ ^]^ We observe good separation quality when particles still have on average a distance slightly bigger than the diameter. This means good separation is achieved at particle concentration range from 0.1 to 1.5 mg mL^−1^ (depending on particle density). Therefore, the proposed method is more suitable for diluted sample conditions and smaller quantities. The latter is typical for samples in the laboratory setting. Furthermore, demonstrated examples for polymer grafted particles are in general weakly phoretically active. Used polymers are neutral but highly water soluble. Since the surfactant contains a positive cationic headgroup, its interaction with negatively charged microgel^[^ ^66^, ^71^ ^]^ and polymer brushes^[^ ^70^ ^]^ would suggest stronger surfactant–polymer interaction in comparison to pNAM and pNIPAM. Thus, we expect the *l*‐LDDO and corresponding phoretic activity to be stronger for anionic polymer brushes and lesser for cationic polyelectrolytes.^[^ ^79^ ^]^ Thus, one may need less separation cycles when separating anionic versus cationic polyelectrolyte brush‐coated particles, or more separation cycles with anionic > neutral > cationic relative to anionic second particle fraction.

## 3 Conclusion

Herein, we introduce a simple method for the separation of different polymer grafted‐ and thermo‐responsive microparticles using microfluidic technology. The principal mechanisms of the separation rely on a light‐induced hovering of chemically active microparticles superimposed in a pressure driven external laminar fluid flow field. The lift‐off tendency depends on the surface morphology of dispersed particles, which provide differences in light‐induced drift motions and then enable a separation of microparticles at different times of retention and sensitive to interfacial properties. To increase the motion contrast between different polymer grafted particles we used a complementary double stimulus, that is, the combination of (I) heating and (II) light illumination. Heating from the bottom interface of the microfluidic channel results in a temperature gradient facing toward the bulk solution, which provides an additional symmetry break closely located to the interface. It amplifies the overall phoretic activity (≈hovering tendency) of the microparticles. Additionally, heating to 45 °C causes structural changes of thermo‐responsive pNIPAM brushes, effectively changing the interfacial properties of the particles. pNIPAM brushes are collapsed and can be assumed to be more semi‐diffuse in comparison to pNAM, which is swollen and thus more diffuse. We observe a gradual decrease of the light‐induced phoretic activity (≈hovering height) of the particles from hard > semi‐diffuse > diffuse interfacial properties. This enables a fractional separation of particles containing thermo‐responsive or non‐responsive polymer brushes, or uncoated particles. Since the brushes are in the size of few nanometers, the relative overall size of the microparticles can be assumed to be equal. Thus, this method demonstrates a new tool to separate microparticles by only minor differences in interfacial properties.

Overall, we demonstrate a simple and effective tool to separate particles very sensitive to interfacial properties. The method has a simple sample preparation process (mixing particles with photosensitive surfactants solution) and only requires an inexpensive device (Led light source, heating stage, and microfluidic technology).

## 4 Experimental Section

4.1

4.1.1

##### Materials

For chemical synthesis are summarized in the Supporting Information (Section S1.1)

##### Polymer Brush Synthesis

Is summarized in the Supporting Information (see Section S1). We estimated the grafting density of pNAM@SiO_2_ and pNIPAM@SiO_2_ using protocols reported elsewhere,^[^ ^70^, ^79^ ^]^ where the grafting density is for grafted microparticles *σ*_pNAM_ = 0.7 chains nm^−2^ and *σ*_pNIPAM_ = 0.29 chains nm^−2^. See details of estimation in Supporting Information (Section S2).

##### Thermogravimetric Analysis (TGA)

We performed thermal analysis of dried samples containing SiO_2_, pNAM@SiO_2_ and pNIPAM@SiO_2_ microparticles using a TGA/DSC 3+ (Mettler Toledo). In a typical measurement, we heated the sample in a synthetic air environment (steam 50 mL min^−1^) over a temperature range of 25–1000 °C and with a heating rate of 10 K min^−1^.

##### Amino Modified Silica Colloids

*Silica colloids* with amino functionalization (–NH_2_) and a diameter *D* = (3.9 ± 0.2) μm are purchased from micro‐Particles GmbH (Germany). We used the particles without further purification for the surface, modifying them with polymer brushes, and for the flow measurements.

##### Light Responsive Surfactant

We synthesized the azobenzene containing trimethyl‐ammonium bromide surfactant (C_4_‐Azo‐OC_6_TMAB, shortly abbreviated AzoC_6_), following the protocol described elsewhere.^[^ ^36^ ^]^ Briefly, the surfactant (Figure 2a) consists of a spacer of 6 methylene groups between the positively charged trimethyl‐ammonium bromide head group and the azobenzene unit with butyl tail attached. Figure S6 (Supporting Information) shows a characteristic UV‐Vis absorption spectra of the surfactant, recorded at photo‐stationary state under exposure to light at 455 nm wavelength. The *trans*‐isomer (denoted as dark) has a characteristic absorption band (π–π* transition) with a maximum of 351 nm. The spectrum of the *cis*‐isomer is characterized by two absorption bands with maxima at 313 nm (π–π* transition) and 437 nm (n–π* transition). The lifetime of the metastable *cis*‐isomer in dark or in red light (*λ* = 625 nm) is ≈48 h at 23 °C. The critical micellar concentration (CMC) of the *trans* and *cis* isomer is 0.5 mM and 4.0 mM.^[^ ^73^ ^]^

##### Zeta Potential, ζ

Zeta‐potential measurements were performed with a Zetasizer Nano ZS (Malvern Panalytical GmbH). Particles were dispersed in aqueous solution with a typical concentration of 0.1 mg mL^−1^. The electrophoretic mobility is measured with a DTS1070 sample cuvette at room temperature in Millipore water, and calculated by instrument software into the zeta‐potential by applying the Smoluchowski approximation.

##### Sample Preparation for Mixing the Microparticles with Photosensitive Surfactant

After the synthesis polymer‐grafted microparticles have been washed (1. dispersing in solvent, 2. centrifugation (1000 RPM, 1 min) of microparticles and the removal of supernatant, 3. dispersing in solvent) three times in a mixture of dioxane and water (1:1) and finally dispersed in Millipore water. Then the particles were dispersed in aqueous solution with azobenzene containing surfactant, washing them 3x times until finally being dispersed in the photosensitive surfactant solution. We expect to remove free polymer chains, potentially adhered on the microparticles, (via surface forces, depletion, vdW, etc.), through this set of several washing cycles in various solvents and surfactant solution. This is based on strong tendency of polymer–surfactant complexation,^[^ ^68^, ^69^, ^70^, ^71^ ^]^ stabilizing free polymer chains in solution as opposed to adhered at the particles interface. The stabilized free polymer chains will be removed from the mixture by removal of the supernatant. In a typical surfactant mixing procedure, we mixed the particles with aqueous solution of the azobenzene‐containing surfactant (*c* = 10 mM, *c* = 0.01 mol l^−1^) to provide a final surfactant concentration of 2 mM and equilibrated this at least for 24 h before performing the flow measurements.

##### Sample Preparation for Flow Measurements

For all the flow measurements we used a commercial microfluidic flow chamber μ‐slide^VI^ with a glass bottom cover slip (Ibidi GmbH) and a sample volume of 40 μL. The chamber geometry is a rectangular microfluidic chamber with following channel dimensions: *h* = 0.54 mm, *d* = 3.8 mm, *L* = 2.4 cm. When an aqueous dispersion of azobenzene‐containing surfactant and microparticles is injected into the microfluidic chamber, the particle sediment settles down to the glass bottom. The chamber is connected to a syringe pump (Havard Apparatus). We kept all samples in dark or in red light to prevent unwanted photo‐isomerization. The flow measurements are conducted at room temperature of *T* = 25 °C.

##### Heating of Microscope Samples

We controlled the temperature using a homemade heating stage.^[^ ^67^ ^]^ The heating stage is coated with an indium tin oxide (ITO) coated glass slide, connected to two silver electrodes. The ITO layer was facing the bottom glass of the sample (microfluidic channel). We calibrated the applied potential to the electrodes in a voltage range of 0–30 V to precisely heat the glass slide up to max. of 60 °C by using thermos‐responsive microgels with varying poly acrylic acid (pAA) content.^[^ ^67^ ^]^ We calibrated the temperature sensor by measuring the resistance from a PT100 resistance thermometer attached to the glass slide. The rate of temperature change is 1 °C per 10 s. In a typical measurement we equilibrated the system for 5 min before running the flow measurement.

##### Optical Microscope Measurements

We used an inverted microscope Olympus IX73, equipped with a red LED (*λ* = 625 nm, M625L1‐C1 (Thorlabs GmbH) light source for the measurements. We did data acquisition through video recording by using a CCD camera (Hamamatsu ORCA‐Flash4.0 LT (C11440)) at 30 frames per second and 4 k resolution (2048 × 2048 pixel).

##### Motion Analysis

We processed the raw video data using a software package (Fiji). First, we converted the video into single‐frame images, then we transferred them into 8‐bit grayscale to perform a binary pixel transformation, using the so‐called default threshold algorithm. To minimized background noise, we using the following operational steps from the implemented Fiji software: “Fillholes”, “Erode” and “Dilate”.

We tracked the microparticles with a self‐made python script, where we calculated the location coordinates for each particle and frame, using object detection from as‐identified contours and the center of mass of the black and white images. We calculated the trajectories per frame and particle via minimum distance and we calculated the momentum velocity (frame‐to‐frame) by multiplying the traveled distance with the framerate. We performed a statistical analysis of all individual velocities by calculating the mean, median velocities observed frame by frame applying the standard deviation (see Statistical Analysis Section). The difference in behavior of median and mean thereby allows for detection of potential biases affecting the mean due to extreme outliers. Analysis is done via python software using several software packages: Bokeh, Numpy, Sympy, OpenCV‐python, Matplotlib, Openpyxl, Pandas, SciPy.

##### Statistical Analysis

Recorded average velocity *U* as a function of the time is calculated from the sample size *n* (number of particles) of individual velocity *U*_i_ for each frame by frame. Here we use the arithmetic mean to obtain *U*:

(3) $$U=\frac{1}{n}\left(\sum_{i=1}^{n}U_{i}\right)$$

and corresponding standard deviation *σ* from the square root of the variance for the sample size of *n* − 1:

(4) $$\sigma =\sqrt{\frac{1}{n-1}\sum_{n=i}{\left(U_{i}-\bar{U}\right)}^{2}}$$

All data is treated without evaluation of outliers.

Statistical analysis was done via Python software using several software packages: Bokeh, Numpy, OpenCV‐python, Matplotlib, Openpyxl, Pandas, SciPy.

## Conflict of Interest

The authors declare no conflict of interest.

## Author Contributions

**Marek Bekir** and **Matthias Hartlieb** concepted the work. **Matthias Hartlieb** and **Anahita Heraji Esfahani** performed all synthesis and analysis regarding the polymer brush grafting on the particles. **Nino Lomadze** synthesized the photosensitive surfactant. **Marek Bekir**, **Andreas Taubert**, **Svetlana Santer** and **Matthias Hartlieb** wrote the manuscript. **Daniela Vasquez Munoz**, **Fabian Rohne**, **Isabel Meier**, **Anahita Heraji Esfahani**, **Anne Nitschke**, and **Cevin Braksch** performed measurements and data analysis.

## Supporting information

Supplementary Material

## Data Availability

The data that support the findings of this study are available from the corresponding author upon reasonable request.
