# Supplementary material for: Separation of Surface Grafted Microparticles via Light and Temperature
Source: Small Sci. 2024 Aug 13;4(10):2400146. doi: 10.1002/smsc.202400146 (PMC11935095; doi:10.1002/smsc.202400146)
Supplement: Supplementary file 1 — Supplementary Material [file SMSC-4-2400146-s001.zip › smsc.202400146-sup-0001-suppdata-S1.pdf]

Supporting Information

Separation of Surface Grafted Microparticles via Light and Temperature

Daniela Vasquez-Muñoz,[1] Fabian Rohne,[1] Isabel Meier, [1] Cevin Braksch,[1] Nino Lomadze,[1] Anahita Heraji Esfahani,[2] Anne Nitschke,[2] Andreas Taubert,[2] Svetlana Santer,[1] Matthias Hartlieb,[2, 3] Marek Bekir[1]**

[1] Institute of Physics and Astronomy, University of Potsdam, Karl-Liebknecht-Str. 24-25, 14476 Potsdam, Germany

[2] Institute of Chemistry, University of Potsdam, Karl-Liebknecht-Str. 24-25, 14476 Potsdam, Germany

[3] Fraunhofer Institute for Applied Polymer Research (IAP), Geiselbergstraße 69, 14476, Potsdam, Germany

Correspondence to: matthias.hartlieb@uni-potsdam.de or marek.bekir@uni-potsdam.de

S1. Synthesis

S1.1 Material and Methods

Chemicals were purchased from TCI (Tokyo Chemical Industry), Merck, Carl Roth, Thermo Fisher scientific, Sigma-Aldrich and American Type Culture Collection (ATCC). Dioxane was used in synthesis grade (>99.5%), while water was deionized and purified via a Milli-Q water purification system. Inhibitor from monomers was removed via recrystallization (*N*-isopropyl acrylamide (NIPAM) purity > 98%, TCI) or via a short column containing aluminum oxide (*N*-Acryloyl morpholine NAM) purity > 98%, TCI) before polymerization.
SEC measurements performed with Tetrahydrofuran (THF) as eluent were recorded with parallel RI and UV detection and the stationary phase was a 300 × 8 mm^2 PSS SDV linear M column at room temperature and at a flow of 0.5 mL min^{-1}. Poly(styrene) (PS) was used as calibrations standard. ^{1}H-NMR spectra were recorded on a Bruker AVANCE NEO 400 MHz spectrometer. As solvents $CDCl_3$ or D_2O were used. The residual proton signal of the solvent was used as internal standard and chemical shift values were reported in ppm.

For polymerization at a UVP Hand Lamp (PL Compact UVL-23) was used. The wavelength was 365 nm with a power output of 4 W equivalent to a 9.13 mW cm^{-2} at sample position. Reaction vessels were placed directly in front of the light source. The intensity of light was directly measured at the sample position with a commercial S170C power meter (Thorlabs).

2,5-Dioxo-1-pyrrolidinyl 2-[(ethoxythioxomethyl)thio]-2-methylpropanoate (MeXan-NHS) was synthesized according to literature procedure.[1,2]

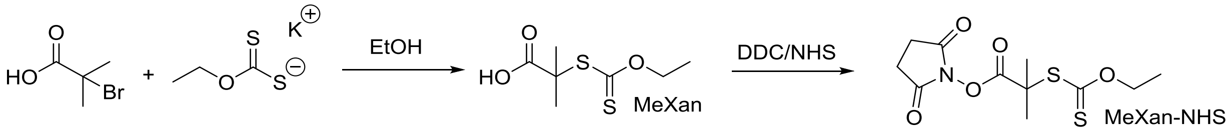

Figure S1. Synthesis of the CTA (MeXan-NHS) used for particle functionalization.

S1.2 Experimental procedures

General procedure for polymerization reactions

For polymerization reactions, monomer (after inhibitor removal) and MeXan-NHS were added to a 5 mL glass vial (Pyrex) and dissolved in a 1:1 mixture of dioxane and water to reach a concentration of 2 mol L^{-1}, and a total volume of 3 mL. Oxygen was removed by purging with nitrogen via a needle for 10 min prior to polymerization.

For particle functionalization, MeXan-NHS was added to an aqueous suspension of Silica microspheres (0.6 mL of a suspension with 5 %wt of particles, corresponding to 0.9 µmol of amino groups on the interface according to supplier information). The mixture was stirred for 2 h before monomer and solvents (amounting to a total 3 mL of a 1:1 mixture of dioxane and water) were added.

In both cases, UV-light (365 nm, 9.13 mW cm^{-2} at sample position) was used, where the vial was placed in front of the light source for predetermined time periods. For each reaction, kinetic investigations were performed collecting samples at 0, 1, 5, 20, and 60 min reaction time to be analyzed via ^{1}H-NMR and SEC. In the case of polymerization in the presence of particles, the samples were centrifuged to remove the silica microsphere before analysis. SEC curves in these cases are of shuttled chains, based on the excess of free CTA in solution.

Table S1. Composition of polymerization mixtures with and without particles.

Sample:	**pNAM**	**pNIPAM**	**pNAM@SiO$_2$**	**pPNIPAM@SiO$_2$**
MeXan	3.7 mg (12 µmol)	3.7 mg (12 µmol)	3.7 mg (12 µmol)	3.7 mg (12 µmol)
NAM	755 µL (6 mmol)	-	755 µL (6 mmol)	-
NIPAM	-	679 mg (6 mmol)	-	679 mg (6 mmol)
Water	1123 µL	1161 µL	523 µL	561 µL
Dioxane	1123 µL	1161 µL	1161 µL	1161 µL
µP-suspension			600 µL	600 µL

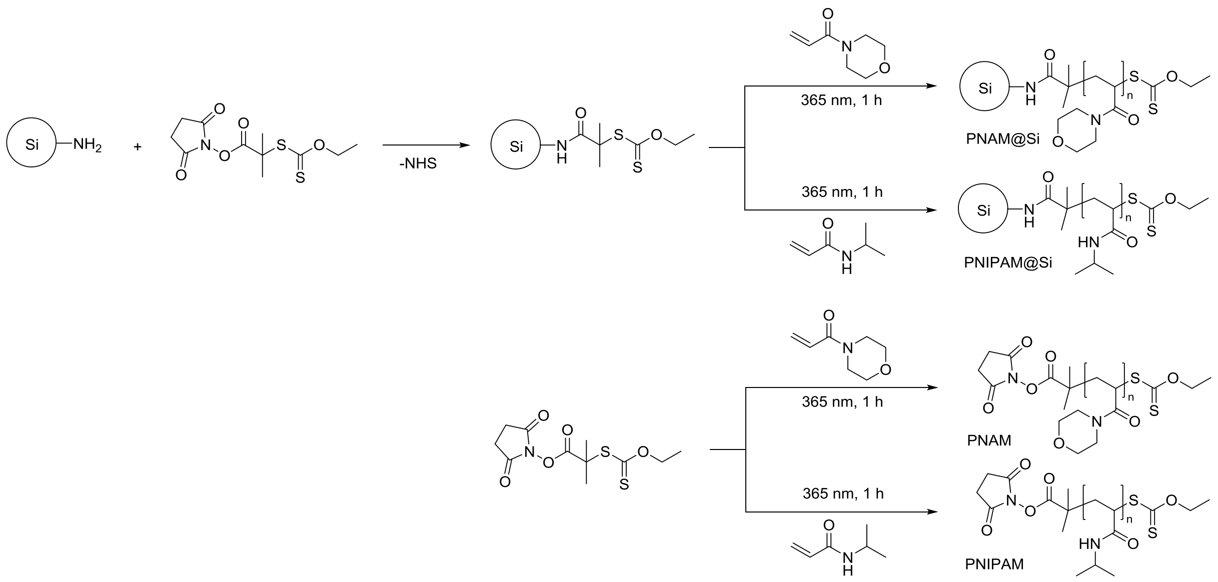

Scheme S1. Schematic representation of polymerization reactions in the presence and absence of silica microspheres.

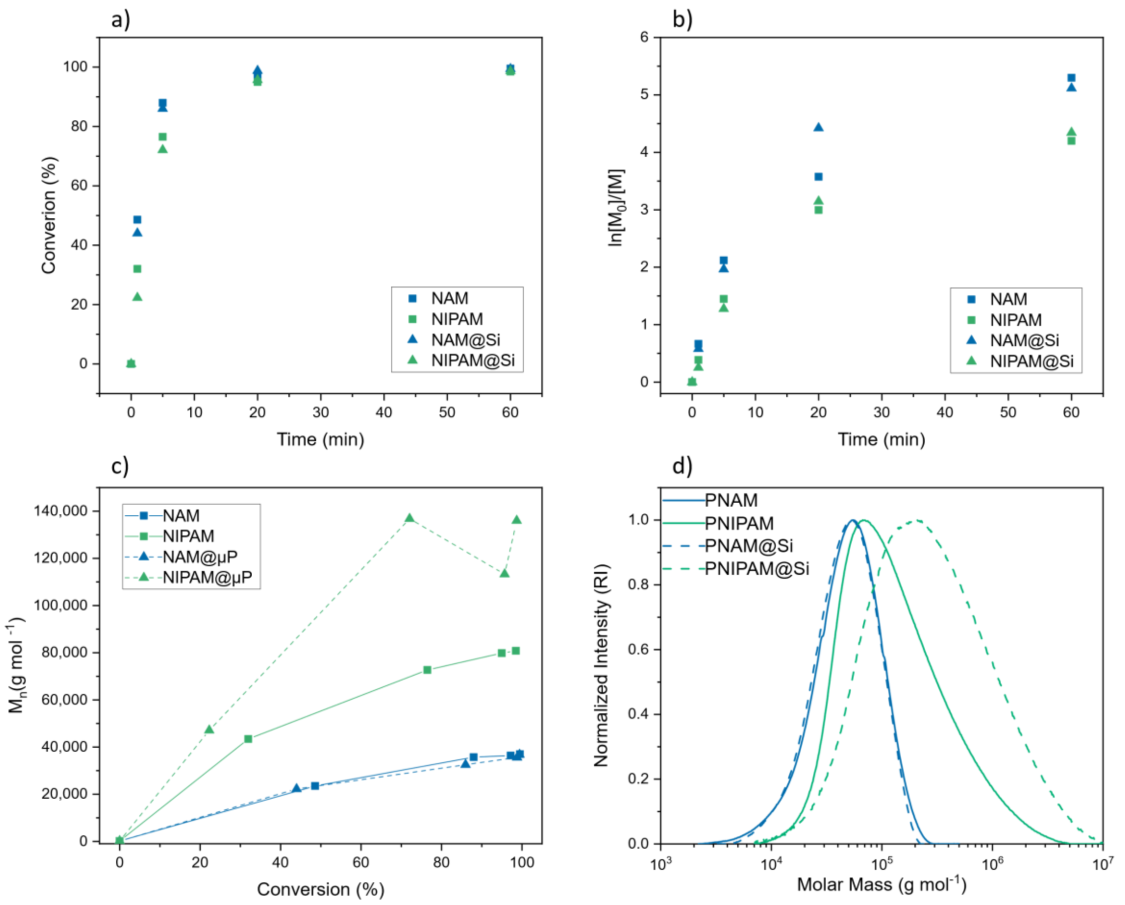

Figure S2. Kinetic investigation of the polymerization of NAM and NIPAM with and without silica microspheres. Monomer conversion (a, b) was determined via 1H-NMR spectroscopy comparing acrylic signals with other signals associated with the monomer. Evolution of molecular weight as a function of conversion (c) was investigated via SEC (THF as eluent, PS calibration). SEC traces of polymers after 60 min are shown in d).

Discussion polymers:

Polymerization reactions were first performed in solution without the presence of silica particles to obtain information about required reaction times with respective combinations of monomer and CTA. As expected, polymerizations proceeded rapidly, reaching close-to quantitative conversion after 20 min. Dispersities were expectedly high due to the low chain transfer coefficients of xanthates with some acrylamides. In particular, PNIPAM shows a tailing at higher molecular weights. It should however be noted that for surface functionalization reactions a high dispersity is not detrimental, as demonstrated in previous studies.[2]

Next, polymerization reactions were carried out in the presence of particles. To enable sufficient attachment of the CTA to the surface prior to polymerization, particles in an aqueous suspension (5 wt%) and MeXan-NHS were left to stir for 2 h before addition of monomer and purging with nitrogen. As the CTA was supplied in a ~12 times excess, a sufficient functionalization of the surface is expected. During polymerization, chain transfer between surface bound chains and polymers growing in solution leads to an exchange of radicals between different sites. Using such a shuttle-CTA approach [3,4] has an additional advantage as chains produced in solution can be analyzed via SEC and their molecular weight distribution should reflect the one of surface bound polymers. While the presence of particles does not affect the size distribution of PNAM, for PNIPAM, a broadening toward higher molecular weights can be observed (**Fig S2d**). It is possible that this behavior is associated with the thermal transition of the polymer in the aqueous medium. As samples were no actively cooled a slight heating by the lamp could induce a phase transition, leading to reduced accessibility of CTA end groups or chain coupling, and hence higher dispersities.[1]

S1.3 Experimental procedures

To confirm the surface functionalization the grafting process was also performed on silica nanoparticles. For this purpose, amino functionalized silica nanospheres (sicastar® from micromod) with a diameter of 50 nm (supplier information were used. The molar amount of amino functionalities was taken from literature, [5] and the amount of particles was adjusted to match the molar amount of amino groups used in the grafting of microparticles. For this 148 µL of a stock solution containing 25 mg/mL of nanoparticles was used. Each step of the reaction was followed by DLS measurements.

Based on the DLS traces (Figure S3) the grafting process can be followed: The initial silica NP have a z-average of around 40 nm and aggregate strongly upon attachment of the CTA. This can be rationalized by the limited water solubility of MeXan, which destabilizes the particles once it accumulates on the surface. The reaction mixture directly after grafting (PNAM@SiO_2 + PNAM) shows two distributions. The smaller one is associated with the formed polymer, as confirmed by a DLS measurement of the separated polymer alone. The second shows the nanoparticles with a substantial increase in size based on the attached shell of polydisperse polymers. After centrifugation, the amount of free shuttle polymer can be reduced drastically. It should be noted that here, only one centrifugation step was used for purification. The reason is the strongly increased difficulty of centrifugation based on the small size of the NP. Moreover, also no washing in the presence of surfactant was performed.

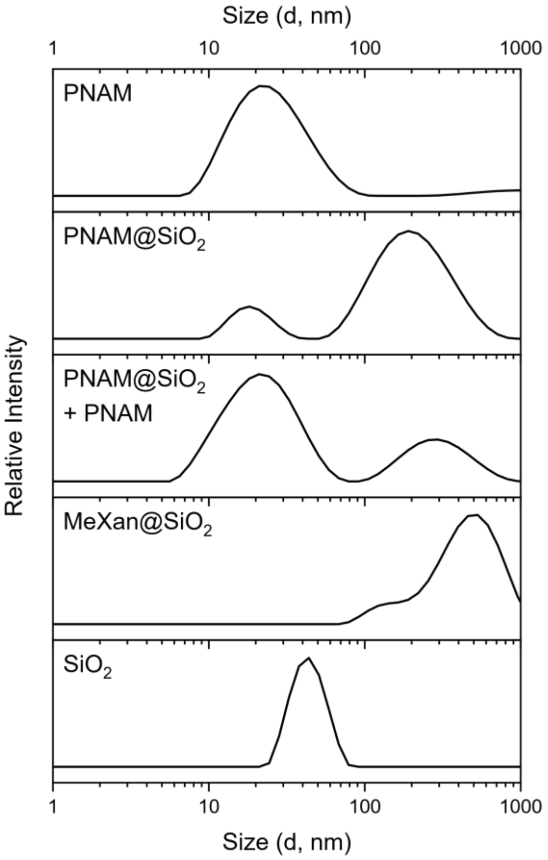

Figures S3: DLS traces of individual steps during NP grafting with PNAM.

Table S2: Site and zeta potential of silica nanoparticles during polymer grafting.

Sample	z-average (nm)	PdI
SiO_2	42	0.035
MeXan@SiO_2	421	0.363
PNAM@SiO_2 + PNAM	27	0.393
PNAM@SiO_2	105	0.607
PNAM	20	0.276

S2. Grafting density estimation

The molecular weight of the silica core, M_{W_C}, is calculated from the volume V_C (average D = 3.9 µm) of the particle multiplying with density ρ_{SiO_2} = 1.8 g/cm^3:

$$M_{W_C} = N_A \cdot \rho_{SiO_2} \cdot V_C = N_A \cdot \rho_{SiO_2} \cdot \frac{4}{3} \cdot \pi \cdot R^3 \,, \qquad \text{S1}$$

with N_A as Avogadro constant, V_C the volume of the silica core.

$$M_{W_C} = 3.36 \cdot 10^{13} \, \frac{\mathrm{g}}{\mathrm{mol}}$$

S2.1. Calculation of the grafting density of polymer@SiO$_2$-brushes

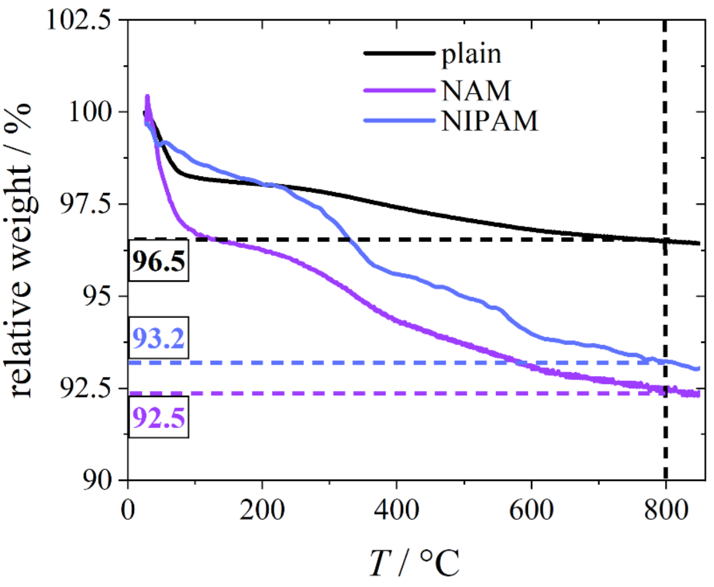

Figure S4. Thermogravimetric analysis data of bare silica particles (SiO_2) and pNIPAM and pNAM coated silica particles (pNIPAM@SiO_2 (blue curve), pNAM@SiO_2 (purple curve)).

First, we determined the weight loss from the pure SiO_2-NPs which can be attributed to the loss of tightly bound water. The difference from the weight loss of silica (SiO_2) compared to pNIPAM@SiO_2 at T = 800 °C is the amount of polymer without the mass of tightly bound water.

$$\Delta m(Polymer) = \Delta m(\mathrm{pNIPAM@SiO_2}, 700\ °\mathrm{C}) - \Delta m(\mathrm{SiO_2}, 700\ °\mathrm{C}) \qquad \text{S2}$$

$$\Delta m(\mathrm{Polymer}) = (100 - 93.2)\% - (100 - 96.5)\% = 6.8\% - 3.5\% = 3.3\%$$

$$M_{W_P} = M_{W_C} \cdot \left(\frac{3.3}{100}\right) = 3.36 \cdot 10^{13} \, \frac{\mathrm{g}}{\mathrm{mol}} \cdot \left(\frac{3.3}{100}\right) = 1.11 \cdot 10^{12} \, \frac{\mathrm{g}}{\mathrm{mol}} \,, \qquad \text{S3}$$

with M_{WP} as the molecular weight of the polymer grafted amount at the silica particle core of the calculated $M_{W,C} = 3.36 \cdot 10^{13}$ g/mol from the known density of 1.8 g/cm^3.

Assuming an average grafted molecular weight per chain of ~40.000 g/mol for pNAM and ~80.000 g/mol for pNIPAM, which is measured from **Figure S2c**, the number of chains per particle N_{chains} can be calculated. Example calculation for pNIPAM:

$$N_{\text{chains}} = \frac{M_{\text{WP}}}{M_{\text{W,C}}} = \frac{3.63 \cdot 10^{12} \frac{\text{g}}{\text{mol}}}{80 \cdot 10^{3} \frac{\text{g}}{\text{mol}}} = 1.39 \cdot 10^{7} \,, \qquad \text{S4}$$

and divided by the surface are of the silica core ($A = 4.78 \cdot 10^7$ nm^2) the grafting density σ is estimated:

$$\sigma = \frac{N_{\text{chains/P}}}{2.29 \cdot 10^{7}\ \text{nm}^2} = 0.29\ \text{nm}^{-2}\,. \qquad \text{S5}$$

Table S3. polymer type, grafted amount of polymer measured via TGA, molecular weight of core-shell structures $M_{W,C+P}$, of pure polymer per particle $M_{W,P}$, of single polymer chain $M_{W,C}$, chains per particle N_{chain}, the grafting density σ

**Polymer type**	**grafted polymer (TGA)**	**$M_{W,C+P}$ (core + polymer)**	**$M_{W,P}$ (polymer per particle)**	**$M_{W,C}$ of single chain**	**N_{chain}**	**σ**
	wt%	g/mol	g/mol	g/mol		1/nm²
pNAM	4.0	$3.52 \cdot 10^{13}$	$1.35 \cdot 10^{12}$	40000	$3.37 \cdot 10^{7}$	0.70
pNIPAM	3.3	$3.49 \cdot 10^{13}$	$1.11 \cdot 10^{12}$	80000	$1.39 \cdot 10^{7}$	0.29

*$M_{W,C} = 3.36 \cdot 10^{13}$ g/mol

S2.2. Supporting experimental data

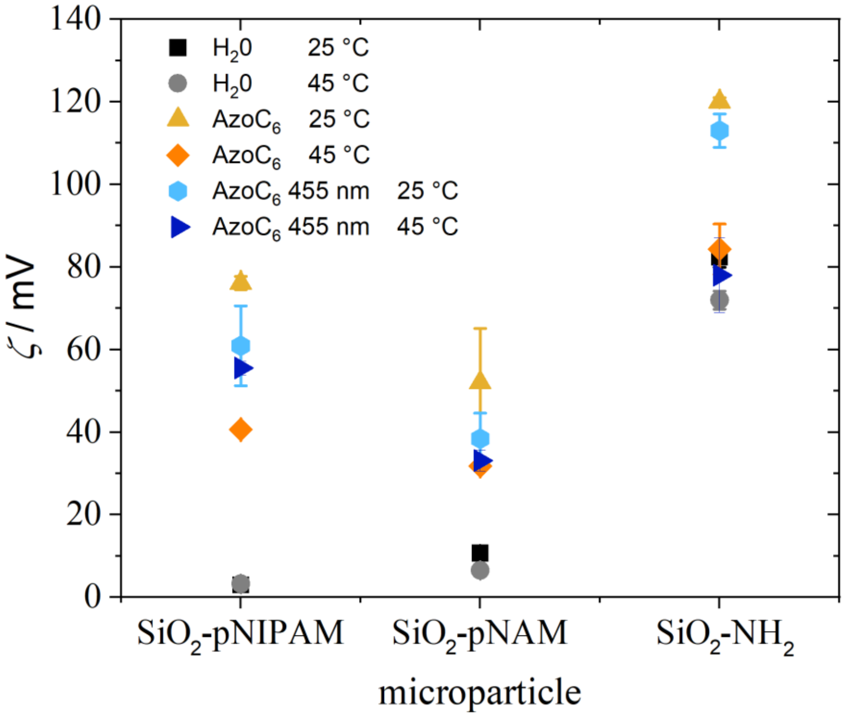

Figure S5. Measured zeta potential as a function of the particle type dispersed in following solution: H_2O – water, $AzoC_6$ – azobenzene containing solution without illumination, $AzoC_6$ 455 nm – azobenzene containing solution after blue light illumination (λ = 455 nm). Data measure either at a temperature of 25 °C or 45 °C.

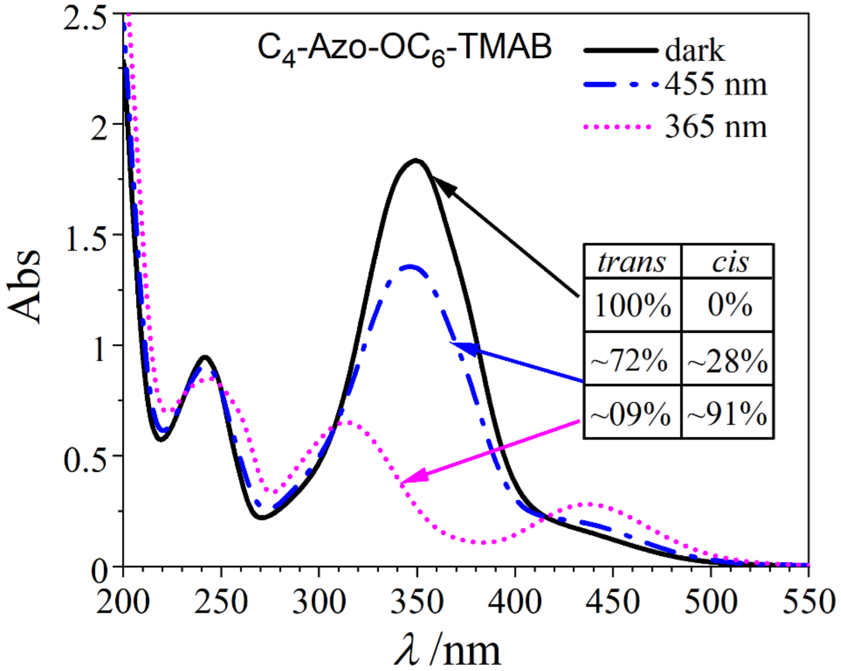

Figure S6. UV-vis spectra of the used azobenzene containing solution measured in a rectangular quartz cuvette of a thickness of 1 cm.

S3. Hovering Height Calculation

S3.1. Used Equations

The velocity scale U_0 is calculated from the experimentally known flow rate[6] $\mathrm{d}V/\mathrm{d}t = 150 \cdot 10^{-9}$ m^3/s as reported by Bekir et. al.:

$$\mathrm{d}V/\mathrm{d}t \sim U_0 \cdot h \cdot l \cdot \left(\frac{1}{12} - \frac{16}{\pi^5} \cdot \frac{\mathrm{h}}{l}\right), \qquad \text{S6}$$

where $l = 2.4$ cm is the length and $h = 0.54$ mm the height of the microfluidic rectangular channel.

The particle elevation to the surface was calculated with the following formular:

$$\frac{U}{a} \approx \mathrm{S} \cdot \frac{h_{\mathrm{ac}}}{a}\left[1 - \frac{5}{16} \cdot \left(\frac{a}{h_{\mathrm{ac}}}\right)^3\right], \qquad \text{S7}$$

where a is the particle radius, h_{ac} the heights above the wall from the center point of the microparticle and S the shear rate which was calculated via:

$$S = \frac{U_0}{2h}. \qquad \text{S8}$$

Note, that the value the particle radiues $a \sim h_{\mathrm{ac}} = 1.95$ µm

S3.1. Calculated Values

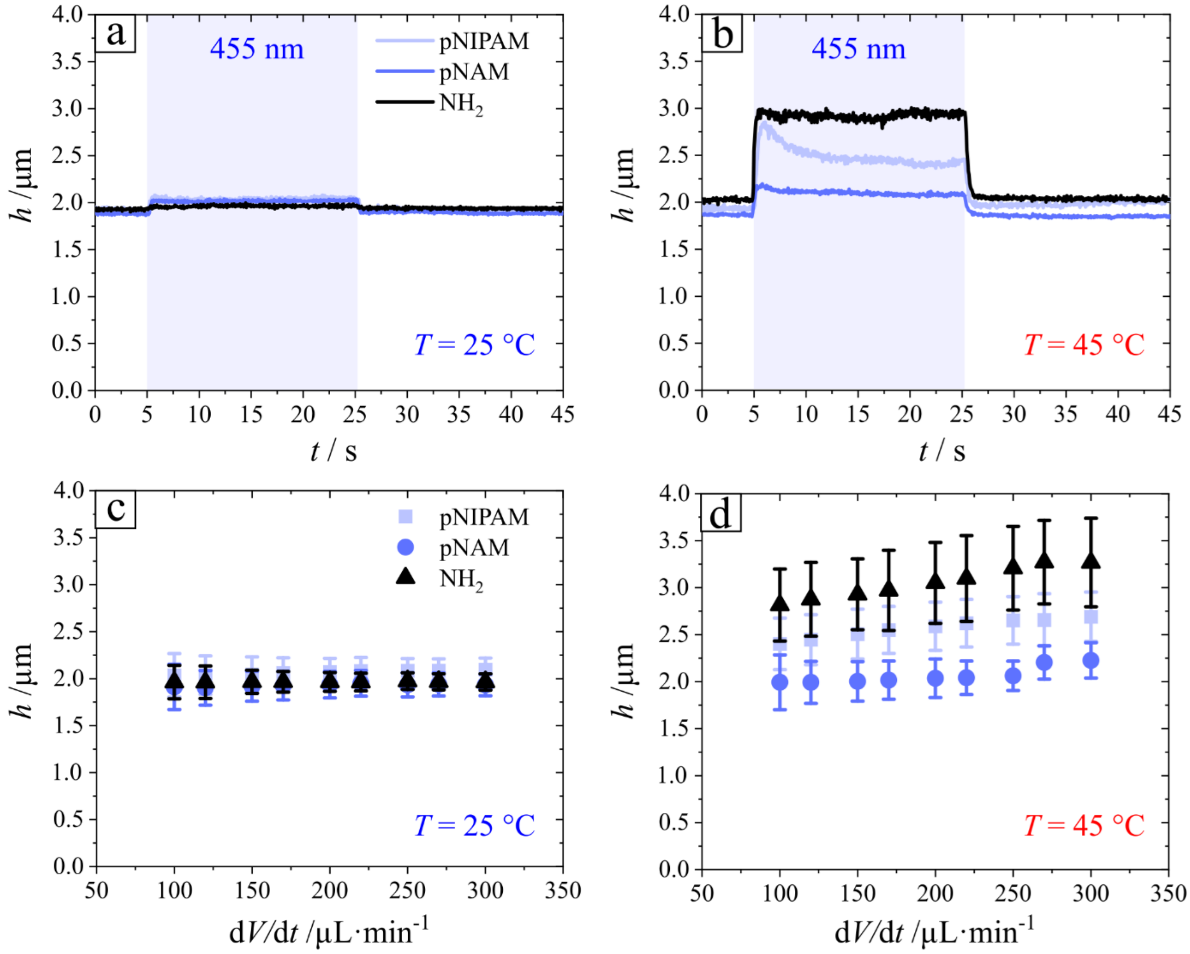

Figure S7. Calculated hovering height h_{ac} using equation from Section S3.1. and data from **Figure 3a,c,d,f** (main article). (a,b) Hovering height as a function of the time (a) at 25 °C (used data **Figure 3a**) and (b) at 45 °C (used data **Figure 3d**). (c,d) Hovering height as a function of the flow rate under illumination (a) at 25 °C (used data **Figure 3c**) and (d) at 45 °C (used data **Figure 3f**).

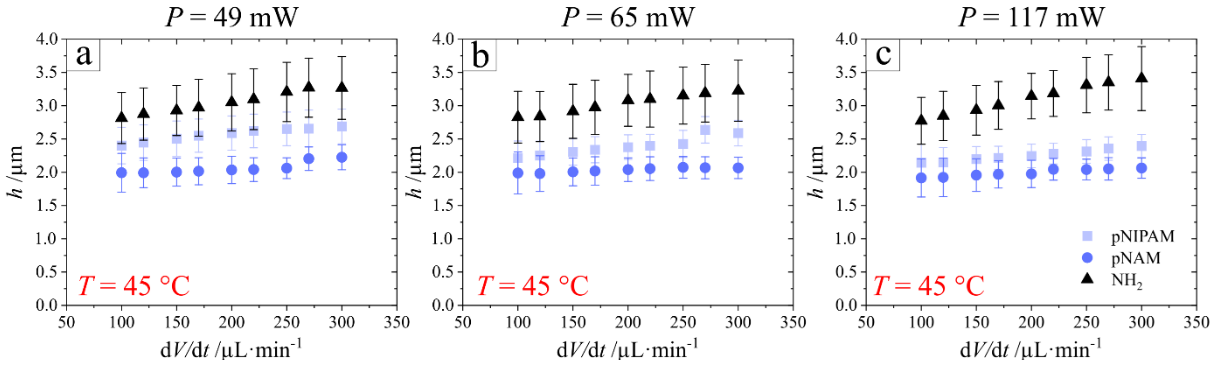

Figure S8. Calculated hovering height using equation from Section S3.1. and data from **Figure 5** (main article). (a,b) Hovering height as a function of the time (a) at 25 °C (used data **Figure 3a**) and (b) at 45 °C (used data **Figure 3d**). (c,d) Hovering height as a function of the flow rate under illumination (a) at 25 °C (used data **Figure 3c**) and (d) at 45 °C (used data **Figure 3f**).

S4. Details of example display regarding particle separation

S4.2. Measurement explanation

This section describes the details of the measurement condition to display the effectiveness of particle separation for the mixture NH_2@SiO_2 and pNAM@SiO_2 microparticles.

For that we mixed NH_2@SiO_2 and pNAM@SiO_2 silica microparticles in a ratio 3:1 with diameter of 4 µm and with fluoresceine-surfactant solution. Fluorescein and cationic surfactant are forming a complex, which yields above the CMC into an emission enhancement of the dye (**Figure S7a**). More anionic fluoresceine accumulated/adsorb on the more positive interface of NH_2@SiO_2 in comparison to pNAM@SiO_2, which yields to a stronger emission signal under recording in FITC-channel (**Figure S7b**). We injected the dispersion into the microfluidic channel and positioned the objective near the inlet position, to be sure, that no other particles can enter the recording area during fluid flow.

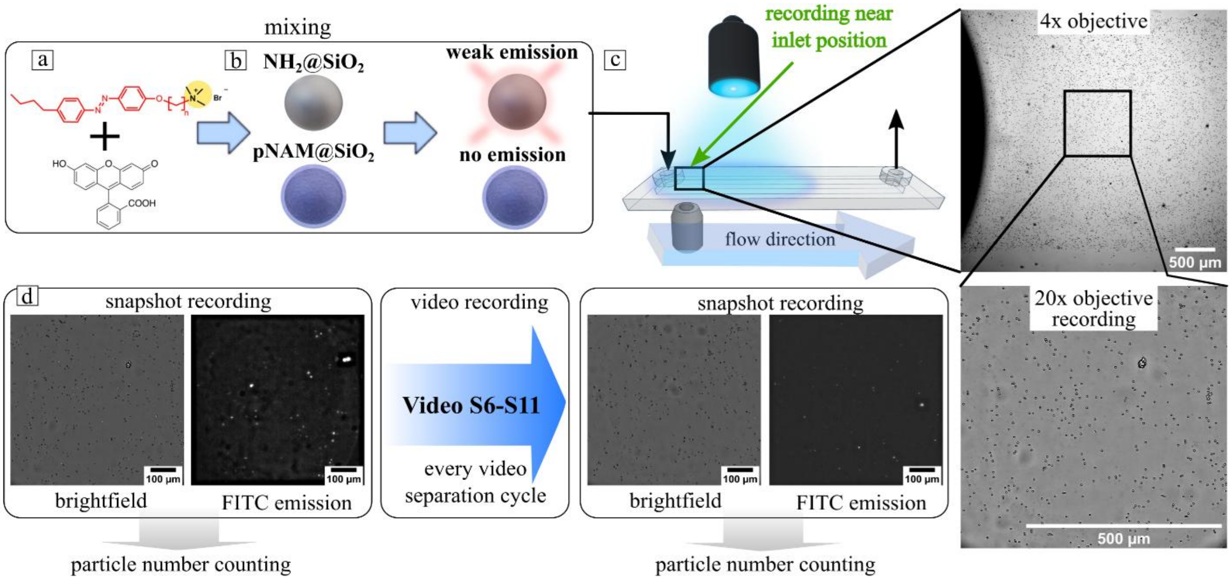

Figure S9. Overall illustration of measurement conditions to display separation effectiveness. (a) Mixing fluoresceine (c = 15 µM) with surfactant solution (c = 1 mM) with (b) NH_2@SiO_2 and pNAM@SiO_2 microparticles (D = 4 µm, $c_{particles,total}$ = 0.5 mg/mL, ratio NH_2/pNAM = 3/1). (c) The dispersion is injected into the microfluidic channel, where the data recording is performed near the inlet position. This is displayed with overall image (4x magnification). Real data recording, counting and analysis is done with 20x magnification in the centre of the image with 4x magnification. Data analysis can only be performed with 20x magnification due to weak emission limitations of NH_2@SiO_2 microparticles. (d) Principal illustration of measurement performance from a series of snapshots in combination with video recording. Before the video recording snapshots are taken to count the microparticles in brightfield and emission signal. Then video recording was performed in forward flow with light (λ = 455 nm) plus heating and reverse flow without light plus heating. **This is one separation cycle**. After that again snapshots are recorded. The snapshot series is displayed from **Figure S8**–**S9** and summarized in **Figure S10**.

To display the effectiveness of separation we measured a series of 5 separation cycles. Every cycle includes a video file (**Videos S6–S11)** in combination of snapshots (6 snapshot pairs) in bright field and FITC emission recording.

Snapshot recording:

Every snapshot is recorded between the video recording. From the snapshots, the number of total particles in the bright field image and that of the $NH_2@SiO_2$ microparticles in the FITC emission image are counted. This allows us to distinguish between the ratio of $NH_2@SiO_2$ and $pNAM@SiO_2$ microparticles after every separation cycle from video file (**Videos S6–S11).**

For details see Supporting Information **Section S4.3**. The snapshot series is displayed from as raw images in **Figure S10–S11** and summarized from data treatment for subsequent particle counting in **Figure S13**.

Video recording (Separation Cycle):

Typical separation cycle: A typical separation cycle in one experimental video show a forward flow under heating (T = 45 °C) and light illumination with blue light (λ = 455 nm, P = 117 mW) for 45 s followed by a reverse flow without light illumination for 45 s. This leads into faster movement of $NH_2@SiO_2$ versus $pNAM@SiO_2$ microparticles in forward direction and thus into superposition difference between both particle types, while in the reverse direction both particles are equally fast — the relative superposition between both types then are equal.

The combination of multiple forward (light) and reverse (without light) cycles under heating yields into spatial separation in the recorded area of both particle types as evidence from counted particles discussed in Supporting Information Section S4.3.

Here five separation cycles have been performed. Every cycle is shown in one video file (**Videos S6–S11**).

S4.2. Raw images of snapshots before experimental videos

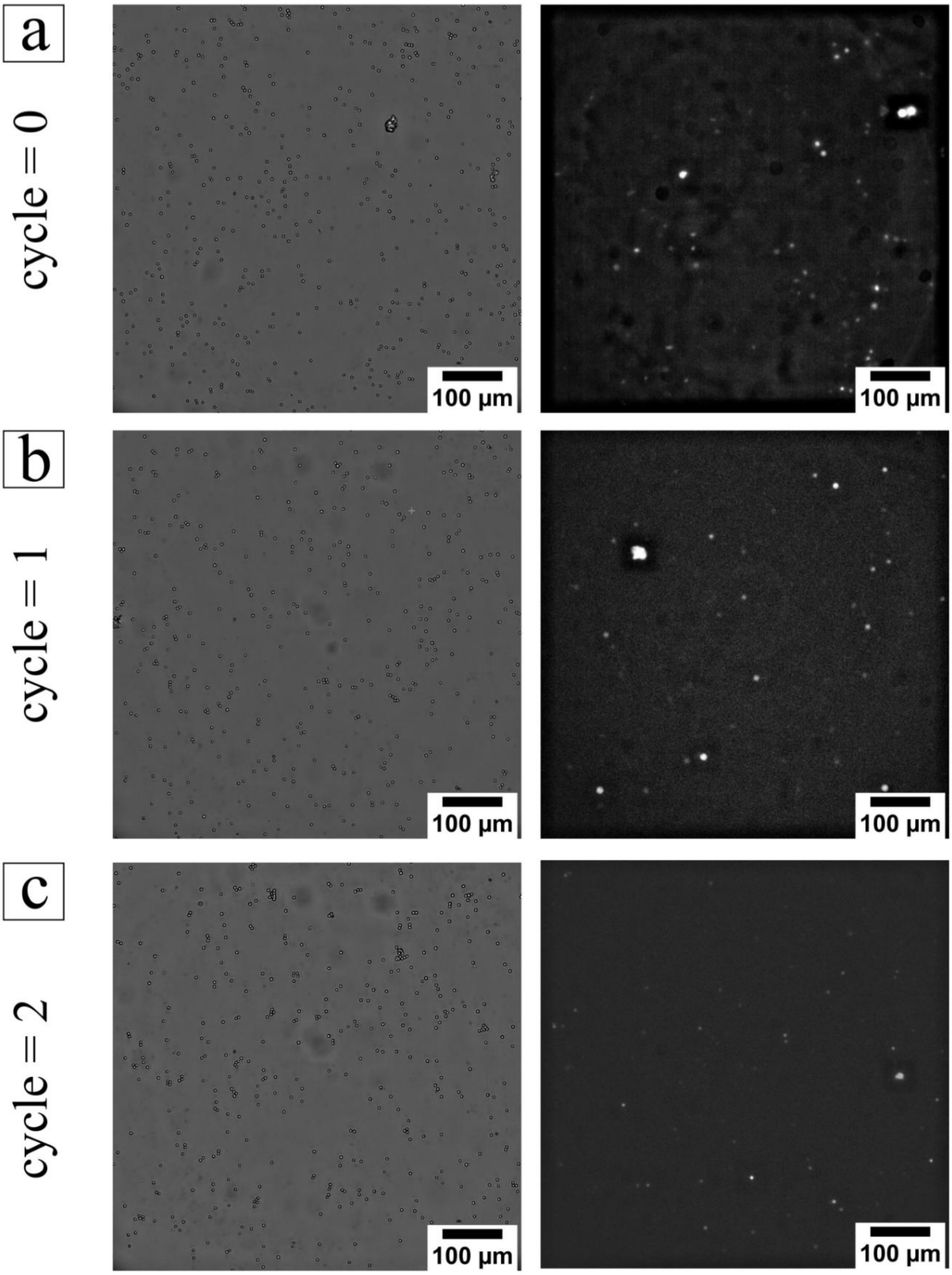

Figure S10. Bright field (left) and emission images (right, gaussian emission intensity of bulk solution background corrected, see experimental data treatment) of local particle mixture of NH_2@SiO_2 and pNAM@SiO_2 microparticle (*D* = 5 µm) immersed in azobenzene containing solution in complex with fluoresceine dye. Snapshot collected near the inlet position of the microfluidic channel. Scale bar is set to 100 µm. Data collected before separation cycle (a) 0, (b) 1, (c) 2.

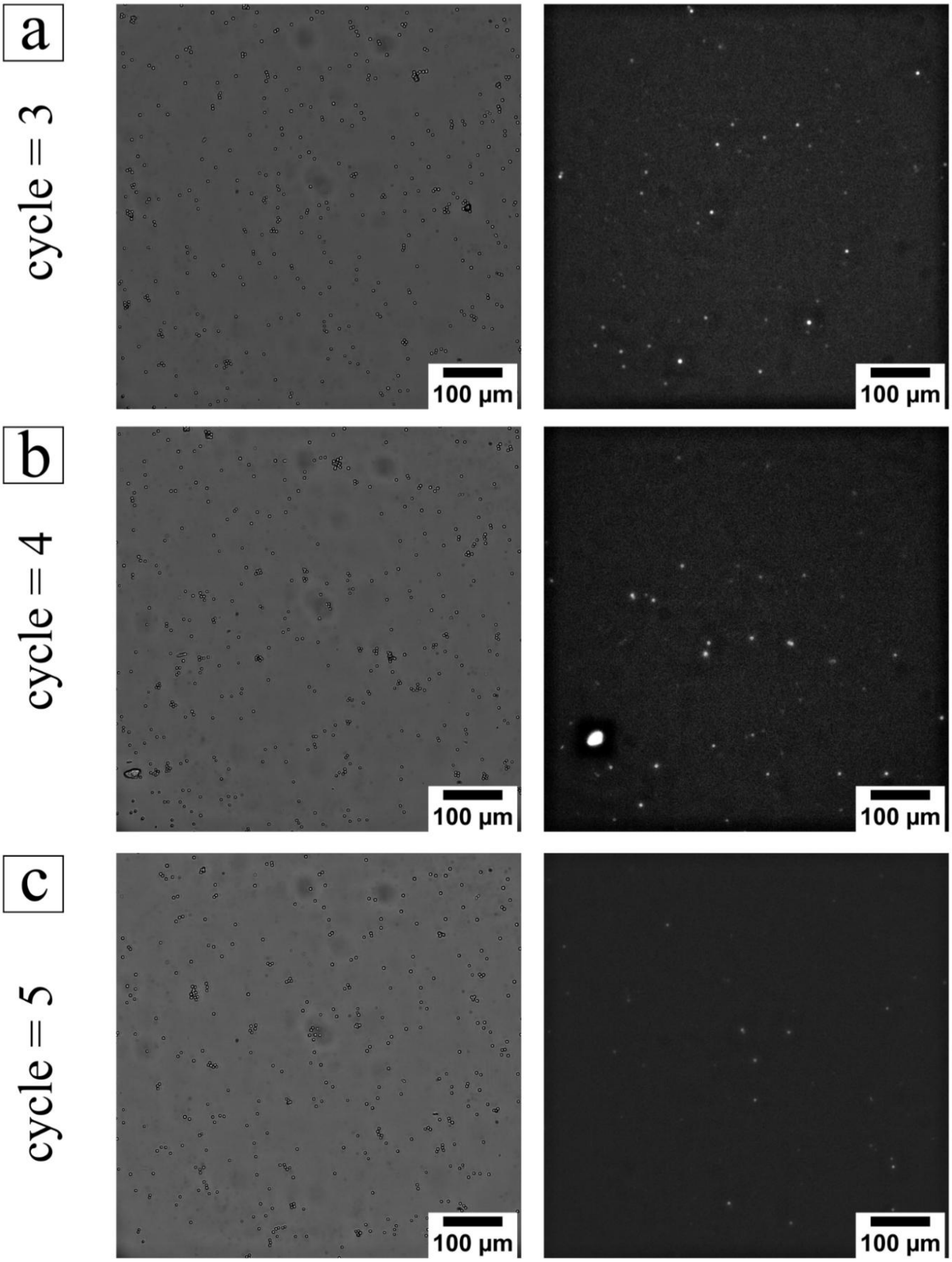

Figure S11. Bright field (left) and emission images (right, gaussian emission intensity of bulk solution background corrected, see experimental data treatment) of local particle mixture of NH_2@SiO_2 and pNAM@SiO_2 microparticle (D = 5 µm) immersed in azobenzene containing solution in complex with fluoresceine dye. Snapshot collected near the inlet position of the microfluidic channel. Scale bar is set to 100 µm. Data collected before separation cycle (a) 3, (b) 4, (c) 5.

S4.3. Experimental data treatment

To calculate the number of microparticles in the brightfield and emission image, prior all images were calculated into a binary pixel information (Thresholding procedure). Always, according to better illumination conditions with red light (λ = 625 nm) the bright field images was easier to threshold. Thus, we show an example treatment for emission image (separation cycle = 0), due to the emission images were in general more difficult to threshold.
Data treatment was done with the Fiji software without needing special plugin packages.

We treated the data in following guideline:
This step was crucial for the images recorded with the FITC channel. Due to the fluoresceine is in complex with the surfactant,[7,8] plenty of fluorescein remain dissolved in the bulk solution and not adsorbed at the particle´s interface. Thus, in a typical emission image, the intensity of the emission has a Gaussian profile with luminescing NH_2@SiO_2 microparticles. An example is displayed in **Figure S12a**. To remove the Gaussian intensity profile the background is manipulated using **Step 1** to obtain a corrected emission image displayed in **Figure S10b** and **Figure S8,9**:

1. **Spatial background contrast manipulation with "Normalized Local Contrast" algorithm**
    a. Set Parameters: Block radius x and y: 80 pixels
    b. Standard deviation: 4.00
    c. Center on
    d. Stretch off
    e. Adjust Brightness and Contrast until emitting particles are fully visible

From the background corrected images the thresholding procedure and counting was started (**Step 2-6**):

2. **Image thresholding with "Yen algorithm", default set**
3. **The binary pixel information is treated with Binary Options standard plugin:**
    a. Do: Dilate
    b. Iterations: 2 / Counts 3 (for increase all black pixels, noise and particles, bigger pixel points increase stronger)
4. **The binary pixel information is treated with Binary Options standard plugin:**
    a. Do: Erode

b. Iterations: 3 / Counts 1 (for decrease all black pixels, noise and particles, all pixel points increase with same amount → removes small pixel points → noise reduction)

5. **The binary pixel information is treated with Binary Options standard plugin:**
   a. Do: Dilate
   b. Iterations: 2 / Counts 3 (for increase all black pixels, noise and particles, bigger pixel points increase stronger → the remaining particles)
6. **The binary pixel information is treated with Binary Options standard plugin:**
   a. Do: Watershed

This yields finally a clear threshold image displayed in **Figure S10c**. Then the number of particles is calculated via:

7. **The black points (particles) are calculated with Analyse Particles standard plugin:**
   a. Size in pixel^2: 3–1200 → (to neglect noise)

Since the size was not necessary, we only count the number of particles.

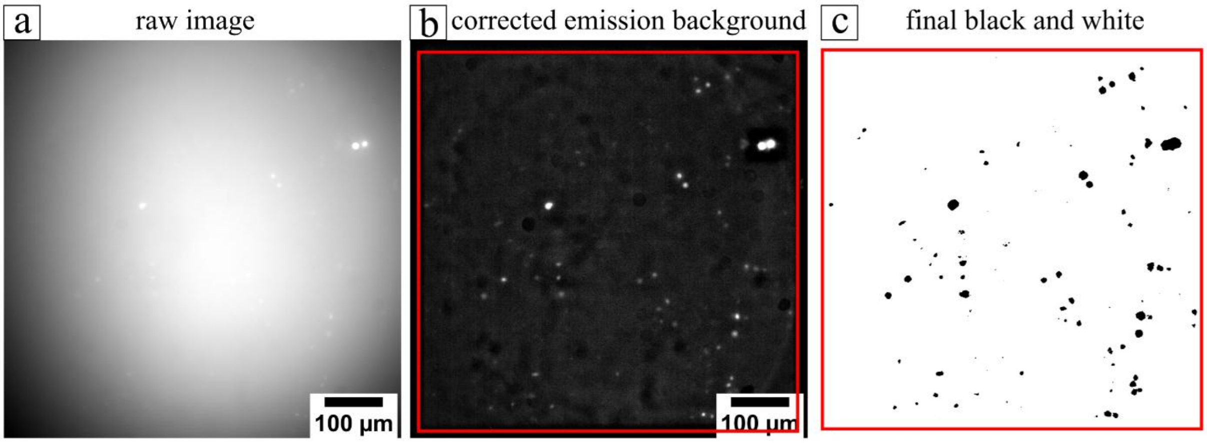

Figure S12. Example image for displaying image calculation into binary pixel information for emission image. Data displayed for separation cycle = 0. (a) recorded raw image. (b) data treated to remove the gaussian emission intensity image from the lamp and dissolved fluoresceine in bulk solution. Data treatment: Step 1. Red area marks the cropping of the data. (c) Threshold image following steps from 2–6.

Due to natural low emission intensity of microparticles (low surface area) and high background emission intensity (surfactant dye complex present in bulk solution) the number of particles might not precisely determined. Here we list following possibilities for the limits of particle number calculations, which yield into less counted particles:

1. **Emitting particle aggregates count as one particle**: During data treatment procedure adjacent emitting particles may be counted as one (overlapping intensity profile plus increased sensitivity of emission signal from data treatment)
2. **Low particle emission to background intensity**: Very weakly emitting particles may be below the emission intensity profile and count as noise.
3. **Removed emission intensity from edges of the image due to data treatment in Step 1**: Particles at the edge of the image **Figure S12b** are removed due to the intensity is average to next neighbouring intensity. At the edges the intensity is always zero, thus the average intensity near at the edge is wrongly determined.
4. **The size of the particles in emission image is bigger determined than actually is, due to the data processing and emission profile of the microparticle strongly manipulate the size**. Steps 2-6 over representing bigger pixel areas, with gaining tendency with increasing pixel area (bigger particles and particle aggregates). Further the emission is over representing the size of the particles, too. The combination of all effects yields to generally bigger sized particles seen in emission in comparison to transmission images.

S4.3. Particle counting

From snapshots displayed in **Figure S13** the binary mixture of NH_2@SiO_2 and pNAM@SiO_2 particles (adjusted ratio of 3:1) are counted after every cycle (**Videos S6–S11**). The NH_2@SiO_2 particles are weakly labelled fluoresceine, while pNAM@SiO_2 particles are unlabeled. From the brightfield image the total number of particles N_{total} are known. The emission image only reveals the number of NH_2@SiO_2 N_{NH2}. With this knowledge we calculated the number of pNAM@SiO_2 N_{pNAM} via:

$$N_{\text{pNAM}} = N_{\text{total}} - N_{\text{NH2}} \,, \qquad \text{S9}$$

where with the relation $N_{\text{total}} = N_{\text{bright field image}}$ and $N_{\text{NH2}} = N_{\text{emission}}$ equation S9 can be rewritten into:

$$N_{\text{SiO}_2} = N_{\text{bright field image}} - N_{\text{emission}} \,. \qquad \text{S10}$$

Data in **Figure S13g** shows the values of N_{total} and N_{NH2} per separation cycle. To calculate the ratio of NH_2@SiO_2 and pNAM@SiO_2 microparticle, we use following equation:

$$\frac{N_{\text{NH}_2}}{N_{\text{pNAM}}} = \frac{N_{\text{emission}}}{N_{\text{bright field image}} - N_{\text{emission}}} \,. \qquad \text{S11}$$

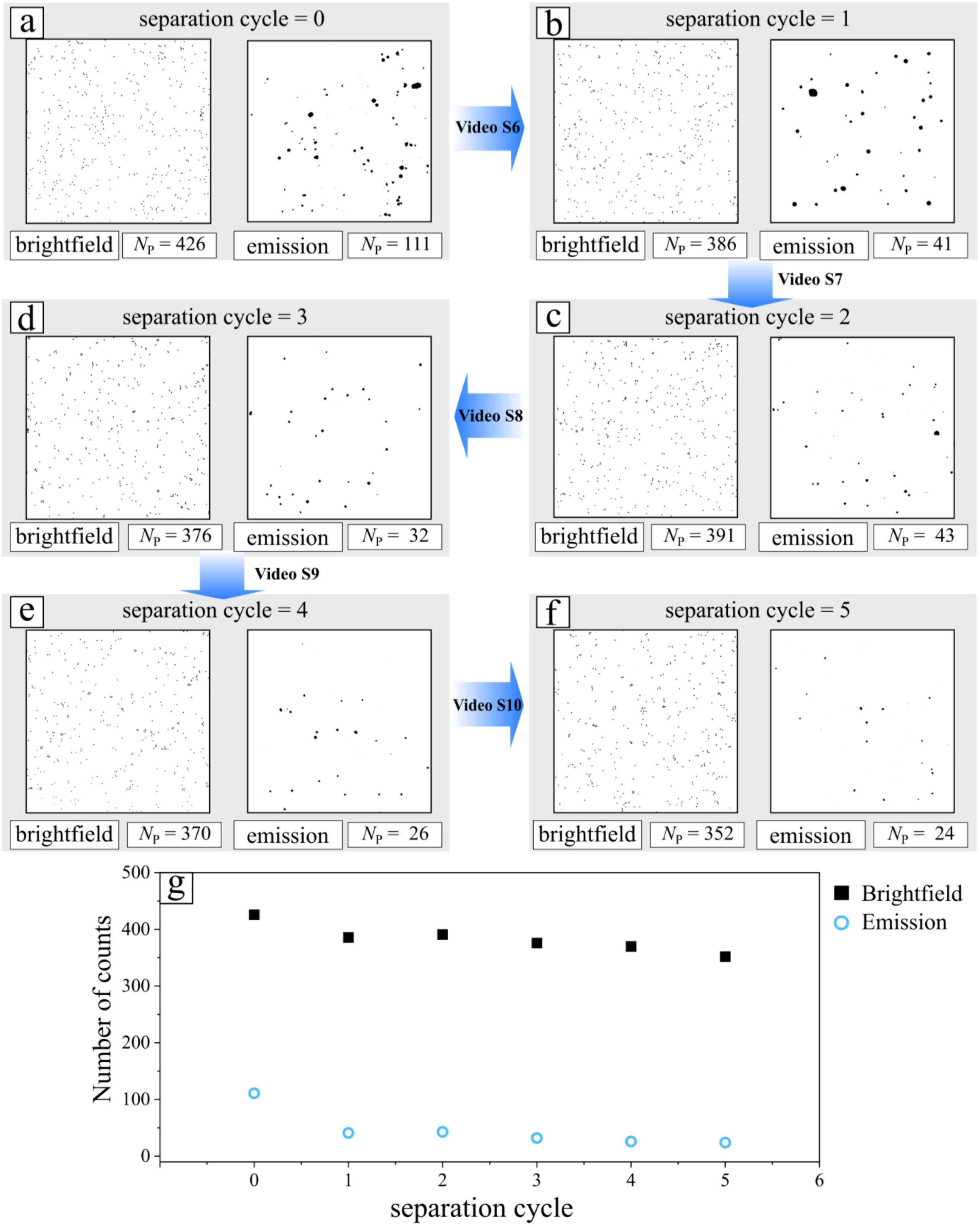

Figure S13. Threshold images (pair) of brightfield (left) and emission (right) recording for subsequent particle number N_P calculation (step 5), (a) separation cycle = 0, (b) separation cycle = 1, (c) separation cycle = 2, (d) separation cycle = 3, (e) separation cycle = 4. (f) separation cycle = 5. Between every step the blue arrow illustrates the used separation cycle with corresponding experimental video file under forward (blue light illumination = 455 nm, I = 11.5 mW/cm^2) and reverse (no light illumination) fluid flow. (f) Counted number of particles in the brightfield and emission images per separation cycle.

S4.4. Particle counting

Further we calculated from values in **Figure S13f** the separation efficiency as a function of the separation cycle. To calculate the separation efficiency we used the equation from reported literature:[9]

$$\text{separation efficiency} = 1 - \frac{N_1}{N_{\text{feed}}} = 1 - \frac{N_{\text{emission}}}{N_{\text{brightfield image}}}, \quad \text{S12}$$

with $N_1 = N_{\text{emission}}$ and $N_{\text{feed}} = N_{\text{brightfield image}}$ as the particle number remained and initially in the microfluidic chamber. Since we performed several separations cycle the separation efficiency is calculated for each separation cycle, which is summarized in Table S3.

Table S4. Separation cycle, particle number in emission N_{emission}, particle number in brightfield image $N_{\text{brightfield image}}$, separation efficiency

separation cycle	N_{emission}	$N_{\text{brightfield image}}$	separation efficiency
0	111	426	0.74
1	386	41	0.89
2	391	43	0.89
3	376	32	0.91
4	370	26	0.92
5	352	24	0.93

S5. References Supporting Information

[1] P. Akarsu, S. Reinicke, A.-C. Lehnen, M. Bekir, A. Böker, M. Hartlieb, M. Reifarth. *Small* **2023**, 2301761.

[2] A.-C. Lehnen, S. Hanke, M. Schneider, C. M. L. Radelof, J. Perestrelo, S. Reinicke, M. Reifarth, A. Taubert, K. M. Arndt, M. Hartlieb. Macromol. Rapid Commun. 2023, n/a, 2300408.

[3] Y. Wang, Z. Zheng, Z. Huang, J. Ling. *Polym. Chem.* **2017**, *8*, 2659-2665.

[4] Z. Zheng, J. Ling, A. H. E. Müller. *Macromol. Rapid Commun.* **2014**, 35, 234-241.

[5] I.-L. Hsiao, S. Fritsch-Decker, A. Leidner, M. Al-Rawi, V. Hug, S. Diabaté, S. L. Grage, M. Meffert, T. Stoeger, D. Gerthsen, A. S. Ulrich, C. M. Niemeyer, C. Weiss, *Small* **2019**, 15, 1805400.

[6] M. Bekir, M. Sperling, D. Vasquez-Muñoz, C. Braksch, A. Böker, N. Lomadze, M. N. Popescu, S. Santer, *Adv. Mater.* **2023**, 35, 2300358.

[7] J. K. Salem, I. M. El-Nahhal, S. F. Salama, *Chemical Physics Letters* **2019**, 730, 445–450.

[8] S.K. Ghosh, M. Ali, H. Chatterjee *Chemical Physics Letters* **2013**, 561–562, 147–152.

[9] M. N. Koleva, S. Liu, C. A. Styan, L. G. Papageorgiou, *Comput. Aided Chem. Eng.* **2016**, 38, 2379–2384.
